# Biodegradation of components from an oxidized polyethylene by a *Rhodococcus* strain isolated from the gut of Atlantic salmon

**DOI:** 10.1128/aem.00174-26

**Published:** 2026-07-10

**Authors:** Ronja Marlonsdotter Sandholm, Dave Rojas Calderón, Marcus Torres Hansen, Ravindra R. Chowreddy, Gustav Vaaje-Kolstad, Sabina Leanti La Rosa

**Affiliations:** 1 Faculty of Chemistry, Biotechnology and Food Science, Norwegian University of Life Sciences 56625, As, Norway; 2 Norner Research AS 826124 , Porsgrunn, Norway; Universidad de los Andes, Bogotá, Colombia

**Keywords:** *Rhodococcus*, metabolic potential, alkane degradation, plastic, low-molecular-weight polyethylene, proteomics, network analysis

## Abstract

Polyethylene (PE) is the most produced synthetic polymer and, consequently, a major source of microplastic waste accumulating globally. Exposure to photo- and thermo-oxidative conditions in the environment can promote PE degradation into carbonyl-containing compounds, hydrocarbons, and low-molecular-weight PE (LMWPE). In both marine and freshwater ecosystems, fish, including Atlantic salmon, can ingest PE and its derivatives, creating opportunities for interactions with their gut microbes. Here, we investigated the capacity of a bacterial isolate from the salmon gut, *Rhodococcus* sp002259485 strain ASF-10, to grow on an LMWPE model substrate for partially depolymerized and oxidized PE. Comparative genomic analyses showed that ASF-10 has a smaller genome than other *Rhodococcus* species yet retains conserved functions, including those related to utilization of medium- and long-chain hydrocarbons. In-depth characterization of the substrate following growth with ASF-10 confirmed depletion of alkanes and 2-ketones deriving from LMWPE, whereas the polymeric component remained unchanged. Proteomic analysis identified multiple enzymes likely involved in the degradation of LMWPE derivatives, including an alkane 1-monooxygenase and cytochrome P450 hydroxylases, as well as proteins for the production of biofilm and a surfactant that may enhance accessibility to the substrate. The bacterium was detected as metabolically active in a metatranscriptomic data set derived from freshwater-reared salmon. Collectively, our findings advance the understanding of the ecology and enzymatic mechanisms underlying the utilization of medium- to long-chain alkanes and oxidized variants thereof, that resemble molecules that can occur from abiotic PE degradation, by a fish gut-associated microbe. This metabolic capacity could be harnessed to develop sustainable strategies for bioremediation of LMWPE derivatives.

**IMPORTANCE:**

The widespread presence of plastics in marine and freshwater environments has raised concerns due to their toxicity when ingested by fish. Microbial mechanisms driving the breakdown of microplastic components, such as low-molecular-weight polyethylene (LMWPE) and derivatives, in gut systems remain poorly understood. This study reveals how a bacterium isolated from the gut of salmon, *Rhodococcus* sp002259485 strain ASF-10, metabolizes alkanes and oxidized variants thereof that can result from abiotic PE decomposition. We identified key enzymes that are potentially involved in this process, as well as in the production of biofilm and surfactants that may facilitate access to the substrate. Besides extending the knowledge of the enzymatic basis for the degradation of PE derivatives in gut-associated microbes from aquatic organisms, our results provide a framework that couples advanced compositional characterization of the substrate with omics techniques, offering valuable insight to support future studies aimed at unequivocally identifying microbes and their enzymes implicated in the transformation of PE derivatives.

## INTRODUCTION

Persistent plastic and microplastic (MP) pollution represents a novel niche within the aquatic environment, serving as an attractive substrate for bacterial growth or a favorable surface for microbial colonization and biofilm development in the otherwise open-water systems (1). In recent years, a multitude of studies have explored the marine “plastisphere,” detailing the diversity, composition, and antibiotic profile of the microbial community colonizing plastic surfaces (2, 3). Aquatic animals, including wild and farmed Atlantic salmon (*Salmo salar*), are exposed to MPs through multiple pathways, including uptake via gills, ingestion of contaminated prey, or consumption of polluted feed (4). Additionally, the aquaculture industry relies heavily on plastic materials for both infrastructure and feed packaging. These include polyethylene (PE), polyethylene terephthalate (PET), and polypropylene, which are used to make cages, ropes, cords, and fishing nets, as well as polystyrene (PS) in foam-filled collars for fish cages, polymer-coated nets, plastic bins for harvesting, feed sacks, and foam buoys. In salmon farming, cages and tanks, as well as feeding systems, are primarily built of high-density PE (HDPE; molecular weight of 300,000–500,000 g/mol), while low-density PE (LDPE; molecular weight of 50,000–300,000 g/mol) is used in protective coatings (5–7). UV light, high temperatures, or oxidizing agents can lead to polymer oxidation and depolymerization, resulting in the formation of low-molecular-weight PE (LMWPE; molecular weight of 1,000–50,000 g/mol; chain length of 70–1,400 carbon atoms), as well as oxidized and non-oxidized fragments, such as ketones and alkanes with a chain length of 5–36 carbons (8, 9). While PE and PE derivatives frequently occur in aquaculture environments and can be ingested by fish (10), no studies to date have examined how gut microbes from aquatic animals could actively interact with MP-derived components. This highlights a critical knowledge gap and underscores the need for further research to determine the fate of PE and its derivatives within the salmon digestive system, including characterization of the metabolic potential and expressed functions of microorganisms interacting with these compounds.

Our group has recently combined targeted isolation strategies with state-of-the-art whole-genome sequencing to generate the Salmon Microbial Genome Atlas (SMGA), a culture collection of bacterial isolates from Atlantic salmon with fully sequenced genomes (11). Additional isolation efforts have genotypically characterized a strain taxonomically assigned as *Rhodococcus* sp002259485 strain ASF-10 (hereafter referred to as *Rhodococcus* sp. ASF-10) (12). Access to these well-characterized strains supports research into how salmon gut bacteria process and utilize key nutrients, such as proteins, lipids, and carbohydrates, from feed (12, 13), or possibly interact with “uncommon” polymers in the gut, such as MPs and hydrocarbons.

Among the bacterial genera implicated in aerobic hydrocarbon biodegradation, *Rhodococcus* stands out alongside *Bacillus* and *Pseudomonas* as one of the key contributors to this process (14, 15). *Rhodococcus* spp. have been isolated from a variety of ecosystems, including soil contaminated with hydrocarbons or plastics, waste sites with naturally weathered plastic debris, seawater, and industrial wastewater (14, 16–18). Comparative genomic analysis across the *Rhodococcus* genus has identified a diverse set of oxidative systems, including multicopper oxidases, alkane monooxygenases, cytochrome P450 hydroxylases, laccases, para-nitrobenzylesterases, and carboxylesterases that have been postulated as compatible with the ability to depolymerize plastics with C–C backbones (including PE), those containing heteroatoms in the main chain, and various polyesters (19, 20). Together, these findings highlight the significant potential of *Rhodococcus* spp. to interact with diverse plastic polymers in multiple environmental contexts, raising the possibility that *Rhodococcus* sp. may also play a role in the breakdown of PE or PE derivatives in gut systems of aquatic animals.

In this study, using a combination of bioinformatic approaches, proteomics, and analytical techniques, we unravel the potential molecular mechanisms deployed by *Rhodococcus* sp. ASF-10 for the degradation of an oxidized LMWPE that resembles an abiotically oxidized form of polymeric PE. We provide genomic evidence demonstrating conserved metabolic functions and the presence of genes encoding enzymes linked to alkane degradation in *Rhodococcus* sp. ASF-10, as well as in a multitude of other *Rhodococcus* isolates from different environments, highlighting the genus’s ability to mineralize these compounds. Proteomic analysis and in-depth substrate characterization demonstrated that *Rhodococcus* sp. ASF-10 actively metabolizes derivatives from oxidized LMWPE, while not utilizing the polymeric fraction. Network analysis of the proteomics data showed strong correlations between the consumption of the LMWPE derivatives, the pathways for alkane degradation, uptake, and β-oxidation of fatty acids, as well as the production of biofilm and biosurfactants. Finally, *Rhodococcus* sp. ASF-10 was detected as metabolically active in a metatranscriptomic data set derived from freshwater-reared salmon across three different life stages. Further examination of its metabolism highlighted several enzymatic features related to alkane, 2-ketone, and fatty acid utilization, suggesting a potential role in the turnover of hydrocarbons *in vivo*.

## MATERIALS AND METHODS

### Bacterial strain and growth conditions

*Rhodococcus* sp. ASF-10 was isolated from the gut of Atlantic salmon (*Salmo salar*) fry, an early freshwater stage when the young fish have absorbed their yolk sac and feed independently (12). The strain was routinely cultivated in Tryptone Soy Broth (TSB, Thermo Scientific, CM01219) at 22°C without agitation. Growth experiments were carried out in minimal salts medium (MM) supplemented with 30 mg/mL of either oxidized low-molecular-weight PE (LMWPE, M_w_ of ~4,000 g/mol, Sigma-Aldrich, 427772), sodium succinate (Sigma-Aldrich, S2378), low-density polyethylene (LDPE FT5230, M_w_ of 97,300 g/mol, Borealis, Austria), tricosane (Sigma-Aldrich, 263850), or 2-hexadecanone (Sigma-Aldrich, 374180). Each liter of MM (pH 7.2) contained 100 mL 10× M9 Medium (0.56 M Na_2_HPO_4_, 0.29 M KH_2_PO_4_, 85.56 mM NaCl, and 93.47 mM NH_4_Cl), 10 mL 100× trace elements solution (17.11 mM EDTA, pH 7.5, 3.07 mM FeCl_3_ × 6H_2_O, 0.62 mM ZnCl_2_, 76.26 µM CuCl_2_ × 2H_2_O, 42.03 µM CoCl_2_ × 6H_2_O, 0.16 mM H_3_BO_3_, and 8.08 µM MnCl_2_ × 4H_2_O), 1 mL 1 M MgSO_4_, 0.3 mL 1 M CaCl_2_, 1 mL 1 mg/mL biotin, and 1 mL 1 mg/mL thiamin. *Rhodococcus* sp. ASF-10 was initially inoculated from a 20% glycerol freeze stock into 5 mL TSB. A 200 µL aliquot of overnight culture was used to inoculate 20 mL of MM in 100 mL Erlenmeyer flasks supplemented with the substrate to be tested. These precultures were subcultured at least three times on the same individual substrate to allow cells to adapt to the specific growth substrate before inoculating the final cultures used for growth profiling and proteomic analysis. All cultures were incubated at 22°C without agitation. Growth was assessed by measuring the optical density (absorbance) at 600 nm (OD_600_) at regular intervals for 10 days. Growth experiments were conducted in two biological replicates, each with three technical replicates.

### Phylogenomic analysis of *Rhodococcus* sp. ASF-10

For comparative genomic analysis of *Rhodococcus* sp. ASF-10, an approach similar to that described by Rong et al. (18) was used, using publicly available genome sequences from 20 representatives of the genus *Rhodococcus*, including members previously reported to be involved in the degradation of plastics, hydrocarbons, and their oxidized derivatives (14, 16–18, 21, 22). The following genome sequences were downloaded from NCBI: *Rhodococcus qingshengii* BF1 (GCA_019279115.1), *Rhodococcus qingshengii* CL-05 (GCA_017910955.1), *Rhodococcus qingshengii* TG-1 (GCA_019048925.1), *Rhodococcus qingshengii* JCM15477 (GCA_001646745.1), *Rhodococcus qingshengii* 7B (GCA_015034605.1), *Rhodococcus qingshengii* RL1 (GCA_008306195.1), *Rhodococcus qingshengii* A34 (GCA_031733215.1), *Rhodococcus* sp. C-2 (GCA_027854095.1), *Rhodococcus erythropolis* R138 (GCA_000696675.2), *Rhodococcus globerulus* NBRC14531 (GCA_001894805.1), *Rhodococcus wratislaviensis* NBRC100605 (GCA_000583735.1), *Rhodococcus koreensis* DSM 44498 (GCA_900105905.1), *Rhodococcus jostii* DSM 44719 (GCA_900105375.1), *Rhodococcus opacus* DSM 43205 (GCA_910591545.1), *Rhodococcus opacus* R7 (GCA_000736435.1), *Rhodococcus ruber* C1 (GCA_016804345.1), *Rhodococcus xishaensis* LHW51113 (GCA_004011825.1), *Rhodococcus spelaei* C9-5 (GCA_006704125.1), *Rhodococcus maanshanensis* DSM 44675 (GCA_900109405.1), and *Rhodococcoides trifolii* CCM 7905 (GCA_014635345.1). The genome of *Corynebacterium antarcticum* CCM 8835 (GCA_016595285.2) was used as outgroup. The genome of *Rhodococcus* sp. ASF-10 was obtained from Rojas Calderón et al., available via FigShare (DOI: 10.6084/m9.figshare.30731261) (12). Taxonomic assignment of all genomes was performed using GTDB-Tk v2.4.0 (23) and GTDB database release 220 (24). The phylogenetic tree was generated using the concatenated alignment of 120 ubiquitous single-copy proteins obtained from GTDB-Tk, followed by the tree inference from the multiple sequence alignment with settings “--prot_model WAG --gamma.” The tree was rooted employing *Corynebacterium antarcticum* CCM 8835 (GCA_016595285.2) as an outgroup and visualized using R v4.3.3 (25) and the R packages tidyverse (26), ggtree (27), tidytree (28), and ape (29). For *Rhodococcus* sp. ASF-10 and *R. trifolii* CCM 7905, average nucleotide identity (ANI) values were calculated using FastANI v1.34 (30). Average amino acid identity (AAI) values were determined using FastAAI v0.1.20 (31).

### Comparative genomic and functional trait analysis of *Rhodococcus* genomes

Metabolic complexity across the 21 *Rhodococcus* genomes was assessed using distillR v0.3.0 (32, 33), an R package that transforms raw annotations into genome-inferred functional traits (GIFTs). DistillR incorporates over 300 curated metabolic pathways and modules from the KEGG (34) and MetaCyc (35) databases to quantify microbial metabolic potential based on the relative abundance of pathway-associated genes. For each genome, GIFT-derived metabolic capacity values are averaged to generate a genome-level metric of overall metabolic capacity, hereafter referred to as the metabolic capacity index (MCI). The *C. antarcticum* and *Rhodococcus* genomes were annotated using DRAM v1.5 (36). DRAM annotations for *Rhodococcus* sp. ASF-10 were obtained from Rojas Calderón et al., available via FigShare (DOI: 10.6084/m9.figshare.30731261) (12). DRAM was used to obtain KEGG Orthology (KO) identifiers, which were then mapped to GIFT functional elements, followed by MCI calculations. Genomes were categorized into three classes based on total genome size: small (<6 Mb), medium (6–8 Mb), and large (>8 Mb). To test for significant differences in metabolic complexity across genome size categories, a one-way analysis of variance (ANOVA) with MCI as the dependent variable and genome size category as the independent variable was performed. Post hoc pairwise comparisons were conducted using Tukey’s honestly significant difference (HSD) with a confidence level set to 0.95 and family-wise error rate correction applied to control for multiple comparisons. To visualize metabolic functional profiles, we performed t-distributed stochastic neighbor embedding (t-SNE) analysis on the GIFT functional elements matrix using the R package Rtsne v0.15 (37). Perplexity was set to the minimum of 30 or (n − 1)/3 to accommodate the sample size. All analyses were performed in R v4.3.3 (25), and results were visualized using ggplot2 v3.5.2 (38), cowplot v1.2.0 (39), and pheatmap v1.0.13 (40).

### Genomic potential for the degradation of synthetic and natural polymers

The genome of *Rhodococcus* sp. ASF-10, along with the genomes of the other 20 *Rhodococcus* spp. used for phylogenomic analysis, was screened for 593 unique gene products associated with the degradation of a selection of synthetic and biodegradable polymers, as well as alkanes (Table S1B). The latter substrate was chosen since the products arising from abiotic degradation of synthetic polymers are identical to or resemble alkanes and other forms of hydrocarbons found in nature (both fossil and renewable). The list of these proteins was compiled from the PlasticDB database (41), the Plastics-Active Enzymes Database PAZy (42), the Hydrocarbon Aerobic Degradation Enzymes and Genes HADEG (43), and published literature (16, 44, 45). The target polymers considered in this search included PE, as well as derivatives and oxidized derivatives of PE (alkanes and 2-ketones), PS, polyurethane (PUR), polyamide (PA), PET, polyvinyl alcohol, polyethylene glycol (PEG), polycaprolactone (PCL), polylactic acid (PLA), polyhydroxyalkanoates (PHA), polyethylene furanoate, polybutyl succinate, poly(butylene succinate-co-adipate), polybutylene adipate terephthalate, and natural rubber (NR). The subject database consisted of the predicted proteome of *Rhodococcus* sp. ASF-10, the predicted proteome of the 20 *Rhodococcus* spp. used for genomic analyses, as well as the predicted proteome of *C. antarcticum* CCM 8835. The amino acid sequences of the proteins encoded by 593 genes associated with plastic and hydrocarbon degradation were used as queries to search the subject database using Protein-Protein BLAST v2.16.0+ (46) with settings “-max_target_seqs 593” (number of sequences in the query data set). BlastP hits were filtered for ≥50% identity (pident), ≥75% query coverage (qcovhsp) to reduce weak or spurious hits, while an e-value of ≤1.0e-5 was used to capture the greatest number of potential homologs for exploratory analysis. For each protein, the best remaining hits were retained. Results were visualized using ggplot2 v3.5.2 (38) in R v4.3.3 (25).

### Assessment of LMWPE degradation by *Rhodococcus* sp. ASF-10

Three analytical techniques were used to gain insight into the effects of *Rhodococcus* sp. ASF-10’s growth on LMWPE structure and composition. To prepare samples for analysis, *Rhodococcus* sp. ASF-10 was cultured in triplicate in MM supplemented with 30 mg/mL LMWPE for 7 days at RT (referred to as *Rh*LMWPE). As controls, cell-free MM was incubated with 30 mg/mL LMWPE (referred to as cfLMWPE). Untreated LMWPE (referred to as uLMWPE), which was not incubated in MM, was also included in the analysis. Following incubation, LMWPE powder was separated from the MM by filtration with Whatman 20 µm cellulose filters (Cytiva, USA, Cat. No. WHA10331554). *Rh*LMWPE was washed in three consecutive steps to remove biofilm. In the first step, 500 µL of dH_2_O was added, and the LMWPE was kept in a ThermoMixer (Eppendorf, Germany) at 1,500 rpm for 30 min at RT. This was followed by an ultrasonication step in a Bransonic 3510-DHT Ultrasonic Bath (Emerson, USA) for 10 min, before removing the dH_2_O. The cleaning was then repeated using 500 µL of a 1 M NaOH solution, followed by washing with 500 µL of 70% ethanol, and the washed LMWPE particles were dried in a Concentrator plus (Eppendorf, Germany) at 45°C for 1 h. The control samples, cfLMWPE and uLMWPE, were subjected to identical treatment.

Fourier-transform infrared spectroscopy (FT-IR) was first performed using a Spectrum Two FT-IR spectrometer (PerkinElmer, Waltham, MA, USA) to detect potential variations in functional groups along the polymer backbone. The signals were obtained with 4 cm^−1^ spectral resolution (eight consecutive readings per measurement) in the 4,000–550 cm^−1^ range using Spectrum IR software (PerkinElmer, Waltham, MA, USA).

Size exclusion chromatography (SEC) was performed using a GPC-IR5 system (Polymer Char, Valencia, Spain) to determine changes in the size of the polymer chains. Approximately 4 mg of each sample was dissolved in 8 mL of 1,2,4-trichlorobenzene at 160°C for 3 h. A 200 µL aliquot was injected into the size exclusion chromatography SEC system equipped with four PLgel 20 µm MIXED-A columns (Agilent Technologies, Santa Clara, CA, USA). Analysis was conducted at 150°C using 1,2,4-trichlorobenzene as the mobile phase (flow rate: 1 mL/min) and a high-sensitivity infrared detector. Calibration was performed using polystyrene standards with narrow molecular mass distributions and peak molecular weights (M_peak_) ranging from 1,140 to 7,500,000 g/mol. The weight-average molecular weight (M_w_), number-average molecular mass (M_n_), and molar mass distribution of the materials were determined.

Finally, changes in the amounts of low-molecular-weight compounds (alkanes and ketones) in the samples were determined using gas chromatography-mass spectrometry (GC-MS). For extraction, approximately 100 mg of sample was mixed with 3 mL of ethyl acetate in a 4 mL glass vial, sealed with a PTFE/Silicone septum, and heated at 95°C for 1.5 h. The extract was filtered (0.2 µm Teflon syringe filter) and analyzed using an Agilent 6890N GC with a 5973 MS detector and GERSTEL MPS2 autosampler. Compounds were separated on a Zebron ZB-5MSPlus column (30 m × 250 µm, 0.25 µm film). Oven temperature was ramped from 60°C to 300°C at 10°C/min over 45 min. Helium (grade 6.0) was used as a carrier gas (3 mL/min). Injections were splitless at 250°C; ion source temperature was 230°C; and ionization was by EI at 70 eV. Spectra were acquired from *m*/*z* 33–720. Each sample was run in triplicate, and compounds were identified using the Wiley 11th/NIST 2017 spectra library. Compound quantification was carried out using the reference compounds BHT (butylated hydroxytoluene) and Tinuvin 120 (2′,4′-di-tert-butylphenyl 3,5-di-tert-butyl-4-hydroxybenzoate), which give differences in responses between lower and higher molecular weight components. Specifically, the response of BHT falls in the region of lower molecular weight alkanes and ketones (up to C16), while Tinuvin 120 falls in the range of higher molecular weight alkanes and ketones. The LMWPE samples analyzed were confirmed to contain no compounds chemically similar to Tinuvin 120 or BHT.

### Analysis of *Rhodococcus* sp. ASF-10’s proteome in response to oxidized LMWPE

*Rhodococcus* sp. ASF-10 was grown at 22°C without agitation in triplicate on 20 mL MM in 100 mL Erlenmeyer flasks supplemented with either 20 mg/mL (wt/vol) sodium succinate or 30 mg/mL (wt/vol) LMWPE, respectively, as a sole carbon source. Samples (20 mL) were harvested at the mid-exponential growth phase. Cells were collected from the liquid cultures by centrifugation (4,500 × *g*, 10 min, 4°C) and resuspended in 50 mM Tris-HCl, pH 7.5, 100 mM NaCl, 0.1% (vol/vol) Triton X-100, 200 mM NaCl, 1 mM dithiothreitol (DTT). Lysis was performed using a bead-beating approach, in which glass beads (size ≤ 106 µm, acid washed; Sigma-Aldrich G8772) were added to the samples and cells were disrupted in three 60 s cycles at 6.5 m/s with a FastPrep24 (MP Biomedicals, CA). Cell debris was removed by centrifugation (16,000 × *g*, 10 min, 4°C), and proteins were precipitated with ice-cold trichloroacetic acid, final concentration of 10% (vol/vol), incubated on ice overnight, centrifuged (15,000 × *g*, 15 min, 4°C) to pellet the precipitated proteins and washed with 300 μL ice-cold 0.01 M HCl in 90% acetone. After removal of the supernatant, the protein pellets were air-dried and resuspended in a buffer containing 5% SDS and 50 mM triethylammonium bicarbonate (pH 8.5). Protein digestion was performed using S-Trap Mini Columns (Protifi, Fairport, NY, USA) according to the manufacturer’s instructions. The resulting peptides were analyzed on a nanoLC-MS/MS system, consisting of a nano UHPLC (nanoElute 2, Bruker Daltonics Inc., Bremen, Germany) coupled to a trapped ion mobility spectrometry/quadrupole time of flight mass spectrometer (timsTOF Pro, Bruker Daltonics Inc., Bremen, Germany). Peptides were separated on an Aurora C18 reverse-phase (1.6 µm, 120 Å) 25 cm × 75 µm analytical column with an integrated emitter (IonOpticks, Melbourne, Australia). Mass spectral data were acquired using DataAnalysis v6.1.

MS raw files were processed using the FragPipe v21.1 (with MSFragger v4.0, IonQuant v1.10.12, Philosopher v5.1.0, DIA-NN v1.8.2 beta 8) (47–50) for protein identification and label-free quantification. Protein identification was performed by searching MS and MS/MS spectra against the complete proteome of *Rhodococcus* sp. ASF-10 (5,538 sequences), supplemented with common contaminants (e.g., keratins, trypsin, and bovine serum albumin, 118 sequences). To estimate false discovery rates (FDRs), a decoy database consisting of reversed sequences of all protein entries was included (5,656 sequences). The resulting protein library consisted of 11,312 proteins. Trypsin was specified as the proteolytic enzyme, allowing one missed cleavage. Variable modifications included N-terminal acetylation, methionine oxidation, deamidation of asparagine and glutamine residues, and pyroglutamate formation at N-terminal glutamines, while carbamidomethylation of cysteines was set as a fixed modification. Protein identifications were filtered to achieve a 1% FDR. A protein was considered “present” if detected in at least two of the three biological replicates for at least one substrate. Missing values were imputed from a normal distribution (width: 0.3; downshift: 1.8 standard deviations from the original distribution) using the “total matrix mode.” Differential abundance analysis was conducted with Rstatix v0.7.2 (51) using an unpaired two-tailed Student’s *t*-test with a permutation-based FDR threshold set at 0.05. Figures were generated using R v4.3.3 (25) with the packages ggplot2 v3.5.1 (38) and cowplot v1.1.3 (39).

### Weighted gene co-expression network analysis

A protein network was constructed using weighted gene co-expression network analysis (WGCNA) as an unsupervised method. Briefly, log₂-transformed proteomic data, including imputed missing values, were used to construct the network and identify protein modules using the R package WGCNA v1.73 (52), which employs the random matrix theory approach. The soft-thresholding (β) was selected using the “pickSoftThreshold” function to approximate scale-free topology (β = 20, R2 = 0.864, mean connectivity = 321). An adjacency matrix was computed with networkType = “signed”, transformed into a topological overlap matrix (TOM), and converted to a dissimilarity matrix. Hierarchical clustering of proteins based on TOM dissimilarity was conducted using average linkage, and modules were defined with the cutreeDynamic function with parameters minClusterSize = 30 and deepSplit = 2. Subsequently, closely related modules were merged using the function mergeCloseModules at a cut height of 0.25. Module eigengenes were calculated, clustered, and correlated with growing substrate (sodium succinate or oxidized LMWPE) to assess module-substrate associations. Module significance was estimated by *t*-test and adjusted *P* values (FDR).

Relevant pathways for this study, such as alkane and ketone degradation, fatty acid transporter systems, fatty acid metabolism, biofilm formation, and surfactant production, were identified for proteins annotated with DRAM that had been assigned a KO identifier using the KEGGREST R package (53). The identified proteins in the pathways listed above were intersected with the proteins in the module positively associated with LMWPE using the R package UpSetR (54).

### Metatranscriptomic analysis

Metatranscriptomic data for the gut of Atlantic salmon fry were obtained from Gupta et al. (13). Raw reads were quality filtered using fastp v0.22.0 (55) with standard parameters, and ribosomal RNA was removed with SortMeRNA v4.3.7 (56). The filtered metatranscriptomic data set was pseudo-aligned using kallisto v0.48.0 (57) to the SMGA (11), supplemented with gene annotations of *Rhodococcus* sp. ASF-10. Results were visualized using the R package pheatmap v1.0.13 (40).

## RESULTS AND DISCUSSION

**Fig 1:**
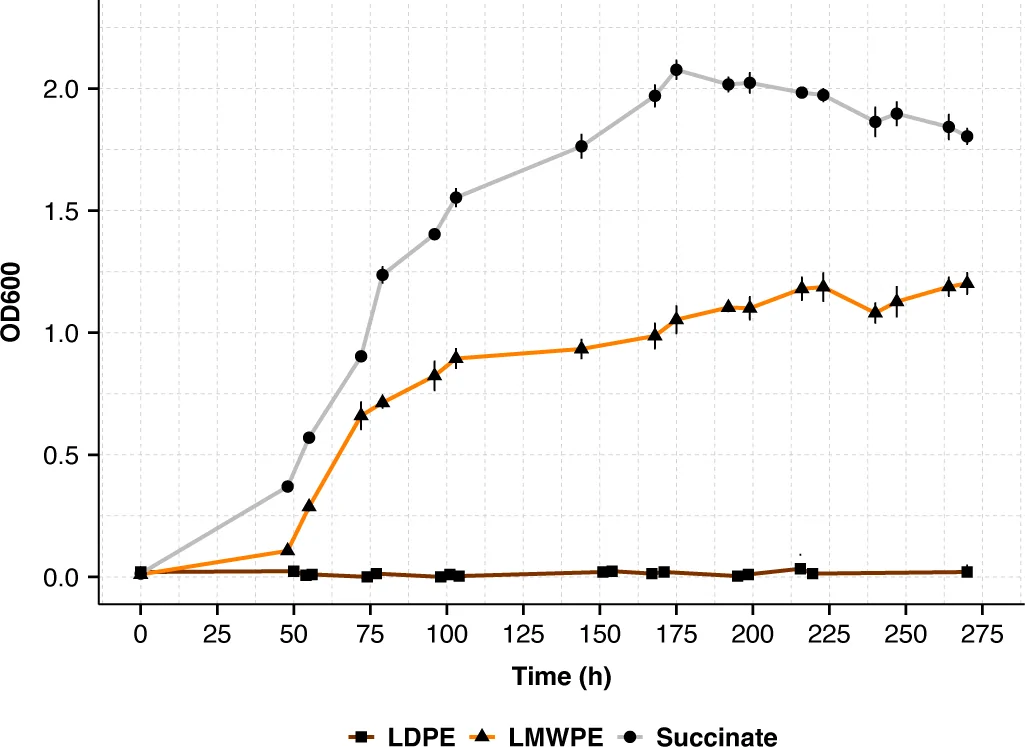
Growth curves for *Rhodococcus* sp. ASF-10 in minimal medium supplemented with LDPE or LMWPE (30 mg/mL) or sodium succinate (20 mg/mL), at 22°C. Growth was measured as the optical density at 600 nm (OD600). The values represent the averages of two biological replicates with technical triplicates. LDPE, low-density polyethylene; LMWPE, low-molecular-weight polyethylene; succinate, sodium succinate.

Bacteria of the genus *Rhodococcus* are frequently detected in 16S rRNA gene amplicon surveys of the gut microbiota of salmon across all developmental stages, from freshwater fry to adults in the marine environment (58). In an effort to identify and characterize salmon gut bacteria interacting with microplastic components that are common in aquaculture, such as PE and degradation products arising from abiotic oxidation of the polymer, we found that *Rhodococcus* sp. ASF-10, isolated from the gut of Atlantic salmon fry (12), displayed the ability to grow on an LMWPE (Fig. 1). This compound was previously shown to be oxidized (59) and can be considered a model substrate for abiotically degraded PE.

### Comparative genomic analysis and functional profiling of representative *Rhodococcus* isolates reveal conserved metabolic traits

To explore the metabolic potential of *Rhodococcus* sp. ASF-10 for possible utilization of plastics, hydrocarbons, and their oxidized derivatives, we first determined the phylogenomic relationship of our isolate and compared its genomic features with those of representative members of the *Rhodococcus* genus (as reported in reference 18), including those previously reported to degrade these compounds. These include *R. qingshengii*, *R. erythropolis*, *R. opacus*, *R. aetherivorans*, and *R. jostii* (22) described to degrade alkanes, and other *Rhodococcus* spp. that are hypothesized to be able to act upon and depolymerize LDPE, including *R. opacus*, *R. ruber*, *R. qingshengii*, and *R. equi* (14, 17, 20, 21).

**Fig 2:**
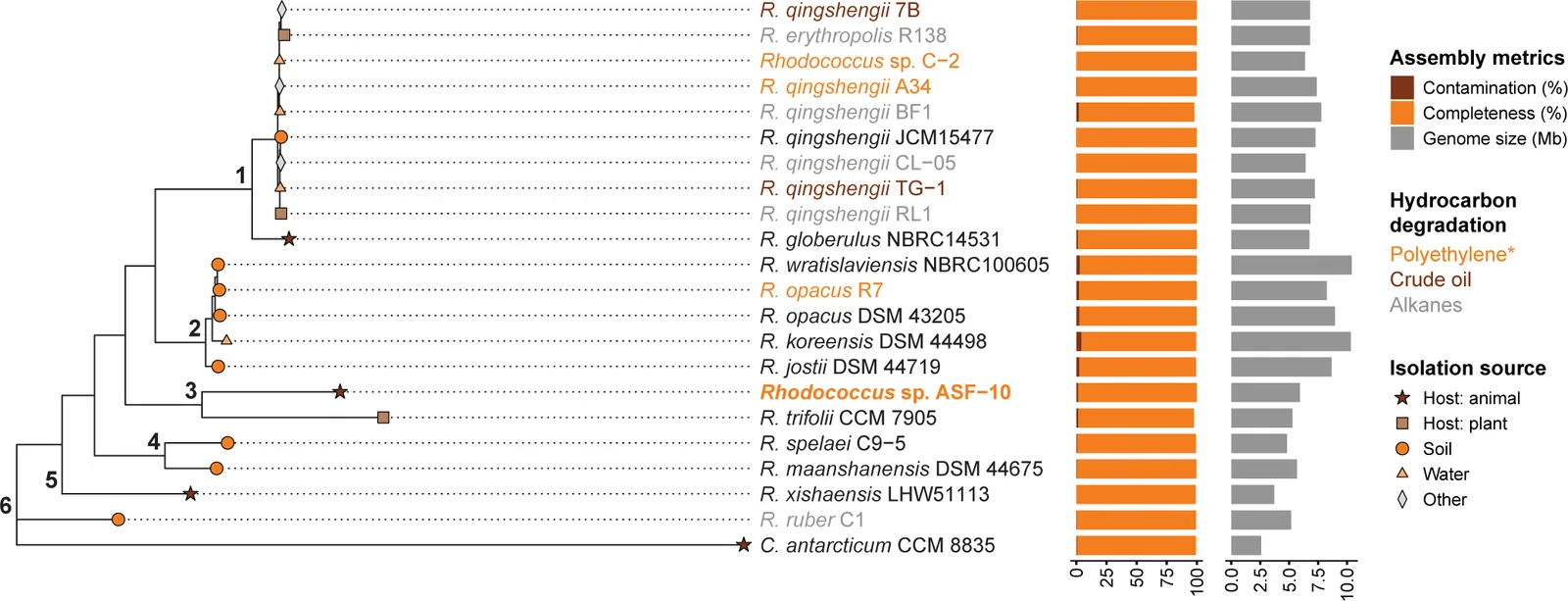
Phylogenomic tree displaying the evolutionary relationships of *Rhodococcus* sp. ASF-10 from this study (highlighted in blue) and 20 publicly available *Rhodococcus* genomes. The genome of *Corynebacterium antarcticum* CCM 8835 (GCA_016595285.2) was used as the outgroup. Numbers (1–6) denote the six clades represented in the tree. Different coloring of the strain names depicts their known ability to degrade or interact with different hydrocarbons (alkanes, oil, and polyethylene). The horizontal bar plots displayed next to each genome depict genome quality (orange bars for completeness and brown bars for contamination), and genome size (gray bars). The * in “oxidized PE/PE” indicates that the substrate used in the studies that characterize this ability in the corresponding bacteria may have contained low-molecular-weight PE derivatives. PE, polyethylene.

To establish the evolutionary relationship between *Rhodococcus* sp. ASF-10 and 20 other members of the genus isolated from a diverse set of environments, a phylogenetic tree based on 120 conserved, single-copy markers shared across the species was constructed, resolving six distinct clades (Fig. 2). *Rhodococcus* sp. ASF-10 exhibited the closest evolutionary relationship to *R. trifolii* CCM 7905, a strain originally isolated from the leaf surface of a plant (60) (clade 3 in Fig. 2). Following comparisons of the two genome sequences, an ANI value of 78.92 and an AAI value of 65.03 indicated that the two species were distantly related. Among the other *Rhodococcus* spp., 10 isolates have previously been associated with either alkane (61), crude oil (62), or PE degradation (16, 18, 21). These include all the *R. qingshengii* strains, except one (*R. qingshengii* JCM15477), and indeed, they all showed a close evolutionary relationship in our analysis (clade 1 in Fig. 2). Clades 2 and 4 in the phylogenomic tree consisted of soil and water isolates of which only *R. opacus* R7 has been reported to be associated with PE utilization (16). Clade 6 included *R. ruber* C1, an isolate known to degrade long-chain alkanes (61). The genomes of the *Rhodococcus* spp. showed variations in genome size, ranging from *R. xishaensis* LHW51113 with a genome of 3.7 Mb to *R. wratislaviensis* NBRC100605 with a genome of 10.4 Mb. Strains within the same clade generally had similar genome sizes, with genomes in clade 2 being between 8.2 and 10.4 Mb (large size genomes), in clade 1 between 6.3 and 7.8 Mb (medium size genomes), and the remaining strains have genomes of <6 Mb (small size genomes). *Rhodococcus* sp. ASF-10 belongs to the group of isolates with small genomes.

**Fig 3:**
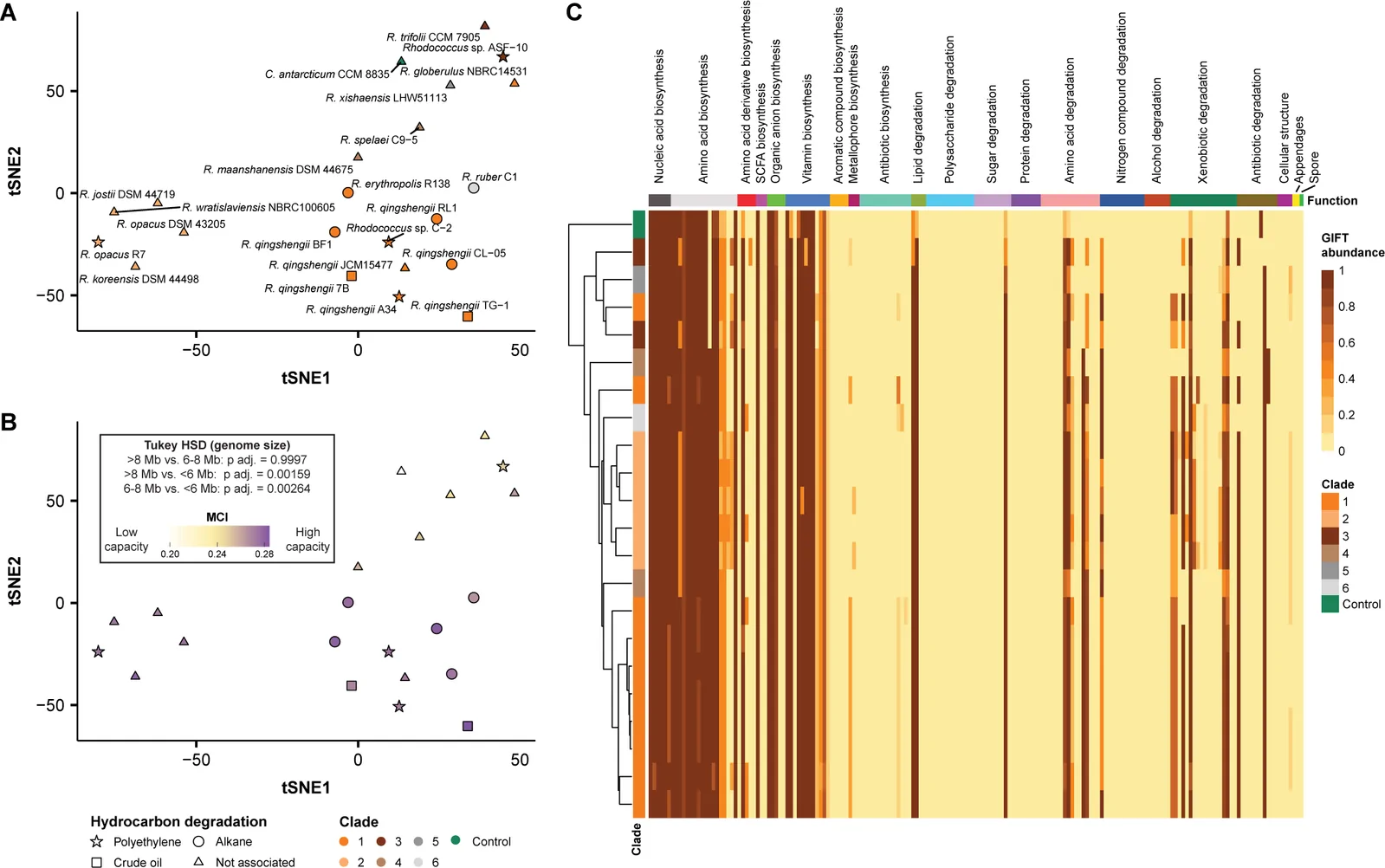
Functional ordination of *Rhodococcus* genomes based on GIFTs and comparison of metabolic trait distributions. (**A**) Ordination of the *Rhodococcus* genomes based on GIFT values using a t-SNE analysis. Genomes are colored according to the clade assignment from the phylogenomic analysis, with *C. antarcticum* CCM 8835 as the control clade. (**B**) Same ordination as in panel A, but genomes are colored by their MCI. Lowest complexity genomes are found in the bottom left, and highest complexity genomes are found in the top right. An ANOVA test and post hoc test (Tukey) were performed to analyze the differences in MCI across strains with different genome sizes. (**C**) Heatmap of GIFT abundance associated with a particular metabolic function (columns) in each *Rhodococcus* genome analyzed in this study. Genomes are clustered based on GIFT abundance (left dendrogram). PE, polyethylene.

To compare specific metabolic capacities encoded by *Rhodococcus* sp. ASF-10 and the 20 selected *Rhodococcus* genomes, we computed the functional potential of each genome based on annotated KEGG Orthology IDs (KO IDs), which were transformed into GIFTs. Genome ordination based on GIFTs values revealed clustering patterns of the *Rhodococcus* species (Fig. 3A and B). Genomes in clade 2 (*R. opacus* spp., *R. wratislaviensis,* and *R. koreensis*) formed a distinct cluster. In addition, genomes in clade 1 (*R. qingshengii* spp.) also grouped closely together. No clear clustering pattern according to the source of isolation was observed. This is consistent with the ecological versatility of *Rhodococcus*, which inhabits diverse environments ranging from soil and water to host-associated niches (63). Species clustering aligned with the clades that were assigned in the phylogenomic analysis (Fig. 2), consistent with the expectation that closely related strains share similar metabolic traits (Fig. 3A). This relationship was also evident when looking at the MCIs of each strain (Fig. 3B). A significant positive relationship was observed between MCI values and genome size. By grouping the genomes by size, an ANOVA and post hoc analysis (Tukey HSD) confirmed that large and medium genomes tend to exhibit higher MCI values than small genomes. Comparisons of large versus small genomes (*P* < 0.01), as well as medium versus small genomes (*P* < 0.01), revealed a highly significant correlation between genome size and metabolic complexity. Genomes with sizes ranging from 6 to 8 Mb and size >8 Mb showed very similar MCIs, with average MCIs of 0.27 ± 0.006 and 0.27 ± 0.003, respectively. Indeed, no significant correlation between large and medium genome sizes and their MCIs was observed for these two groups. Small genomes showed a significantly lower metabolic complexity (average MCI 0.24 ± 0.02) compared to large and medium-sized genomes. The lack of a significant difference in MCI values between medium and large genomes suggests a threshold (~6 Mb in genome size) beyond which further increases in genome size do not confer additional metabolic capacity. This may suggest that core metabolic functions are established by 6 Mb, and that additional genome content beyond 6 Mb could be redundant genes, mobile elements, or adaptive traits that enable survival across varied environments rather than increased metabolic complexity.

Next, we compared the biosynthetic and degradative capacities for specific biomolecules across the studied *Rhodococcus* strains (Fig. 3C). Despite no drastic differences being observed across most pathways, certain metabolic functions varied among clades. Specifically, clade 2 genomes include pathways for amino acid biosynthesis and xenobiotic degradation that are not found in the genomes belonging to the other clades (Fig. 3C). Clade 3, which includes *Rhodococcus* ASF-10 (small genome size), was characterized by the lack of pathways for the production of glutamate, gamma-aminobutyric acid, and vitamin B3 (niacin). Otherwise, the core functions of all *Rhodococcus* genomes are generally the same. They all have the complete pathway for degradation of lipids, specifically fatty acid degradation. Importantly, this pathway includes enzymes for the utilization of alkanes, which seems to be a core function for *Rhodococcus* spp.

Overall, our results underscore that phylogenomic relationships largely govern metabolic trait patterns in the studied *Rhodococcus* genomes, with genome size directly correlated with metabolic complexity up to a threshold of approximately 6 Mb. Remarkably, degradation of fatty acids, and the biosynthesis of multiple nucleic acids (inosinic acid, uracil, cytosine, and adenine) and amino acids (serine, threonine, valine, leucine, proline, tyrosine), as well as vitamin B2 (riboflavin), are conserved among the *Rhodococcus* spp., including *Rhodococcus* ASF-10. Other pathways, which are mostly conserved across the genomes, include the production of the short-chain fatty acid acetate, organic acids (succinate, fumarate, and citrate), several B vitamins, and most amino acids, as well as the degradation of aromatic compounds, such as toluene, benzoate, catechol, and trans-cinnamate.

### Genomic potential for degradation of hydrocarbons in *Rhodococcus* spp.

**Fig 4:**
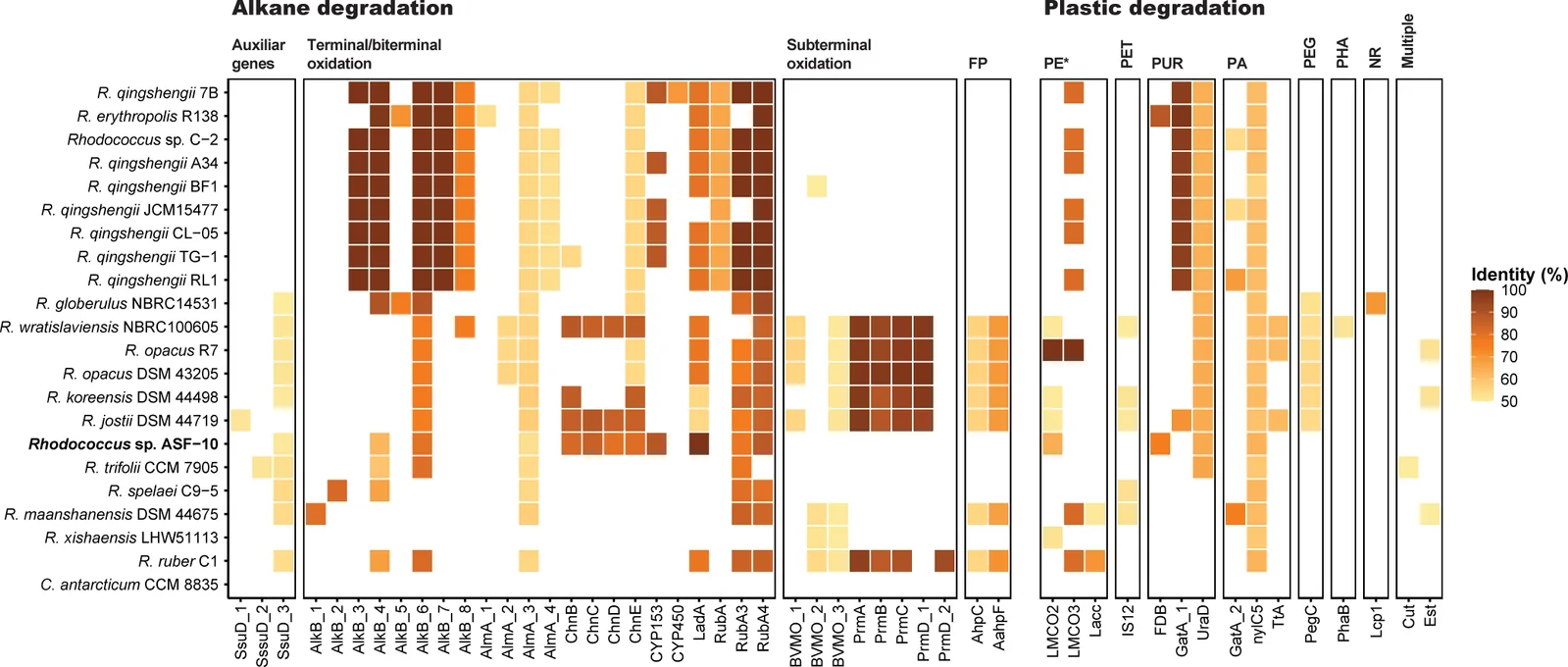
Detection of enzymes for alkane degradation, fossil-based plastics, and biodegradable plastics in the predicted proteome of *Rhodococcus* sp. ASF-10 and 20 publicly available *Rhodococcus* genomes. The predicted proteome of *Corynebacterium antarcticum* CCM 8835 was included as a control in the bioinformatic analysis, as it is known to lack the targeted proteins. Hits represent the best match in each predicted proteome. The * in “PE” (polyethylene) indicates that the substrate used in the studies that characterize this ability in the corresponding bacteria may have contained low-molecular-weight PE derivatives. Polyethylene terephthalate (PET), polyurethane (PUR), polyamide (PA), polyethylene glycol (PEG), polyhydroxyalkanoates (PHA), natural rubber (NR). The panel “multiple” contains hydrolases which act on multiple plastics (the cutinase Cut on PCL/PPA and the lipase Est on PHBV/PHO/PLA/PCL/PES). BlastP hits and metadata from PAZy, HADEG, and PlasticDB are provided in Table S1.

To assess the *Rhodococcus* strains’ potential to utilize hydrocarbons, we generated a reference database that includes 593 unique proteins associated with the utilization of diverse synthetic and biodegradable polymers, as well as alkanes (Table S1B). The list of these proteins was compiled from the PlasticDB database (41), the Plastics-Active Enzymes Database PAZy (42), the Hydrocarbon Aerobic Degradation Enzymes and Genes HADEG (43), and published literature (16, 44, 45). BLAST searches indicated that all *Rhodococcus* predicted proteomes encoded proteins with ≥50% sequence identity to the proteins in the reference databases, while, as expected, no hits were obtained for *C. antarcticum* (Fig. 4 and Table S1A). The capacity to metabolize or interact with alkanes appeared to be broadly conserved across the *Rhodococcus* genus (Fig. 4). In general, closely related strains shared similar potential for alkane degradation. For example, all the *R. qingshengii* strains, along with *R. globerulus* NBRC14531 in clade 1, have alkane 1-monooxygenase (AlkB), long-chain alkane monooxygenase (LadA), and a FAD-binding monooxygenase associated with long-chain alkane degradation (AlmA), as well as the electron-transfer protein rubredoxin (RubA). These enzymes are known to be involved in the initial terminal/biterminal hydroxylation of alkanes (64). Clade 2, consisting of *R. wratislaviensis* NBRC100605, *R. opacus* R7, *R. opacus* DSM 43205, *R. koreensis* DSM 44498, and *R. jostii* DSM 44719 (Fig. 2), have enzymes for both subterminal and terminal/biterminal oxidation of alkanes (Fig. 4). In the predicted proteome of *Rhodococcus* sp. ASF-10, we detected 12 proteins associated with alkane degradation, particularly those involved in terminal/biterminal oxidation of alkanes, and 4 with plastic degradation, specifically PE, PUR, and PA (Table S1A). Overall, the species within the same clade generally have proteins involved in a main degradation mechanism of alkanes, with clades 1, 3, 4, and 5 mainly using terminal/biterminal oxidation, and clades 2 and 6 using subterminal oxidation.

Though 489 of the 593 entries in our database were proteins related to the degradation of synthetic polymers and biopolymers, only 16 plastic-related enzymes were detected in the *Rhodococcus* predicted proteomes (Fig. 4 and Table S1). Some of the *Rhodococcus* spp., including *Rhodococcus* sp. ASF-10 from this study, had hits for two laccase-like multicopper oxidases (LMCOs), LMCO2 and LMCO3, which were previously associated with the degradation of PE (16, 20). Another copper-binding enzyme, a laccase (Lacc) (17), was detected in the strains *R. ruber* C1 and *R. maanshanensis* DSM 44675. Although these enzymes have been linked to the degradation of PE, Lacc has not yet been biochemically confirmed as a PE-degrading enzyme. Notably, the LMCO2 and LMCO3 were characterized using the same LMWPE material used in this study (Sigma-Aldrich, 427772), which has been shown to contain PE derivatives (alkanes) and oxidized PE derivatives (ketones), in addition to the polymeric component of LMWPE (59). This opens the possibility that the two LMCOs could be active only on the alkanes and ketones present in the substrate mixture. All *Rhodococcus* strains previously associated with PE degradation possess putative homologs for one or two of these enzymes (Fig. 4). However, in the absence of experimental evidence confirming actual degradation of the polymeric component in LMWPE, the presence of the genes coding for the laccases and LMCOs does not confirm a true capacity for PE degradation.

Next, we explored the genomic potential of the *Rhodococcus* spp. to interact with other synthetic and biodegradable polymers (Fig. 4). The predicted proteome of *R. wratislaviensis* NBRC100605, *R. koreensis* DSM 44498, *R. jostii* DSM 44719, *R. spelaei* C9-5, and *R. maanshanensis* DSM 44675 includes enzymes for PET degradation (cutinase IS12 [65]). For PUR utilization, *R. erythropolis* R139 and *Rhodococcus* sp. ASF-10 have amidases (FDB [66]), while the putative ability to utilize PUR through the urethanase GatA_1 (66) was detected among all the *R. qingshengii* spp. and *R. erythropolis* R138. Seventeen out of the 21 *Rhodococcus* spp. examined possessed a gene coding a protein similar to the esterase UraD, previously associated with PUR degradation (66). Regarding the utilization of PA, a protein highly similar to the amidase nylC5 for nylon decomposition (67) was detected in all the *Rhodococcus* spp. The protein GatA_2 for utilization of nylon-6 (68) was detected only in the predicted proteome of *Rhodococcus* sp. C-2, *R. qingshengii* JCM15477, and *R. qingshengii* RL1. Finally, the predicted proteome of *R. jostii* DSM 44719, *R. opacus* R7, and *R. wratislaviensis* NBRC100605 included a protein similar to the amidase TtA, previously shown to be involved in nylon-6 degradation (68). The potential ability to interact with PHA through the poly(3-hydroxyalkanoate) depolymerase PhaB (69) was detected only in *R. wratislaviensis* NBRC100605, while *R. globerulus* NBRC14531 was the only bacterium with a protein similar to the rubber oxidase Lcp1 for NR degradation (70). The enzyme PegC for PEG degradation was identified in the predicted proteome of *R. globerulus* NBRC14531, *R. wratislaviensis* NBRC100605, *R. opacus* spp., *R. koreensis* DSM 43205, and *R. jostii* DSM 44719.

Overall, our results show that the *Rhodococcus* strains examined in this study display genomic potential for utilization of alkanes, diverse plastics, and natural polymers, such as PE, PET, PUR, PA, PEG, PHA, and NR, with *Rhodococcus* sp. ASF-10 exhibiting potential for the utilization of alkanes, PE, PUR, and nylon.

### Characterization of LMWPE shows that *Rhodococcus* sp. ASF-10 can only utilize accessible alkanes and oxidized derivatives

**Fig 5:**
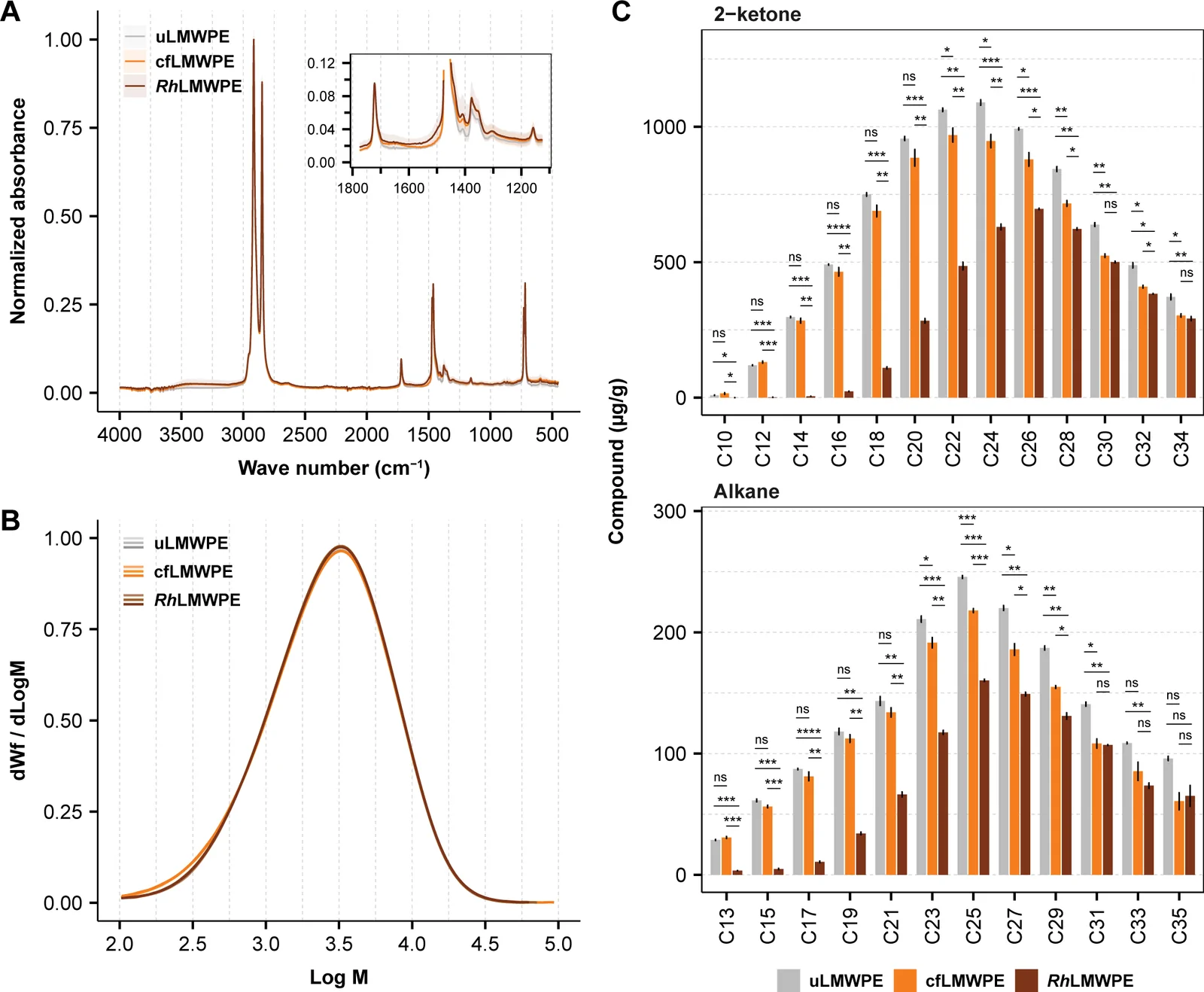
Analyses of LMWPE post-incubation with *Rhodococcus* sp. ASF-10. The isolate was grown with 30 mg/mL on LMWPE in MM (*Rh*LMWPE). Controls consisting of LMWPE incubated in cell-free MM (cfLMWPE) and untreated LMWPE (uLMWPE) were included. (**A**) FT-IR spectra showing the mean normalized absorbance of the samples, with the standard deviation included as shaded areas. (**B**) SEC chromatograms of samples. Note that the chromatograms are superimposing in the high molar mass regions, indicating no degradation of the polymeric fraction of the LMWPE substrate, as any chain scission would be expected to shift the distribution toward lower molar masses (i.e., to the left). (**C**) GC-MS results of LMWPE samples showing the amount of each compound detected in the LMWPE ethyl acetate extracts. Of note, in the materials we analyzed, no odd-numbered carbon 2-ketones or even-numbered carbon alkanes were detected (see Table S2C). The product pattern of alkanes and 2-ketones has been confirmed by fragmentation patterns in EI mode and is a genuine feature of the LMWPE used in this study. Data are averages ± standard deviations (error bars) of three biological replicates, and significance levels are displayed for pairwise *t*-test [not significant (ns), *P* ≤ 0.05 (*), *P* ≤ 0.01 (**), *P* ≤ 0.001 (***), and *P* ≤ 0.0001 (****)]. Experiments were conducted in triplicate. Source data for FT-IR, SEC, and GC-MS can be found in Table S2.

To determine whether *Rhodococcus* sp. ASF-10 can utilize pristine LDPE or an LMWPE with oxidized derivatives, the isolate was cultured in an MM containing either of these substrates as a sole carbon source. *Rhodococcus* sp. ASF-10 grew well on the oxidized LMWPE, reaching an OD600 of approximately 1.0, whereas no growth was observed on LDPE (Fig. 1). Substrate utilization by the isolate was examined by characterizing the LMWPE substrate before and after bacterial growth (Fig. 5 and Table S2). Firstly, we employed FT-IR spectroscopy, which is a commonly used method to determine changes on the surface of the analyzed material, where the formation of carbonyl groups can be detected through the appearance of a peak at ~1,750 cm^−1^. While extensively used to characterize PE post-bacterial growth, FT-IR results are an indirect way to determine surface changes and should be interpreted with caution, as the existence of oxidized compounds such as ketones and aldehydes in the material, or residual protein or biofilm arising following bacterial growth can result in a signal (71, 72). To circumvent this issue, a washing protocol that included ethanol and sodium hydroxide in combination with sonication was employed. LMWPE was confirmed to contain carbonyl groups (59), which were detected in the untreated material, as well as in the LMWPE incubated in MM with and without *Rhodococcus* sp. ASF-10, as indicated by the presence of peaks at ~1,750 cm^−1^ (Fig. 5A). No difference was observed between the experimental samples (*Rh*LMWPE), the cell-free samples (cfLMWPE), or untreated controls (uLMWPE), as no additional peaks appeared post-incubation. Another limitation of FT-IR is that it can only detect changes in the outer layers of LMWPE and may not fully capture chemical changes in the deeper layers or reductions in the high-molecular-weight fraction indicative of PE degradation. To address this, the LMWPE material before and after growth with *Rhodococcus* sp. ASF-10 was also analyzed using SEC. The superimposition of the SEC chromatograms of the *Rh*LMWPE and cfLMWPE samples in the high molecular weight region indicated that no degradation occurred in the polymeric fraction of the material, while differences were observed in the low molecular weight region (LogM 2–2.5; Fig. 5B). To identify the compounds being utilized by *Rhodococcus* sp. ASF-10 during growth, GC-MS analysis was carried out on the LMWPE substrate. Based on the recovery data in Table S2C, a net decrease in recoverable organic material corresponding to a few mg per g LMWPE over 7 days was observed in the presence of *Rhodococcus* ASF-10, consistent with substrate utilization by the bacterium. Indeed, after growth with *Rhodococcus* sp. ASF-10, there was a significant reduction (*P* < 0.05) in the amount of 2-ketones and alkanes, particularly of the shorter chain lengths (Fig. 5C). Overall, under the conditions of this study, *Rhodococcus* sp. ASF-10 was shown to utilize 2-ketones up to 28 carbons and alkanes up to 29 carbons in length.

### *Rhodococcus* sp. ASF-10 metabolizes LMWPE derivatives through alkane and fatty acid utilization pathways

**Fig 6:**
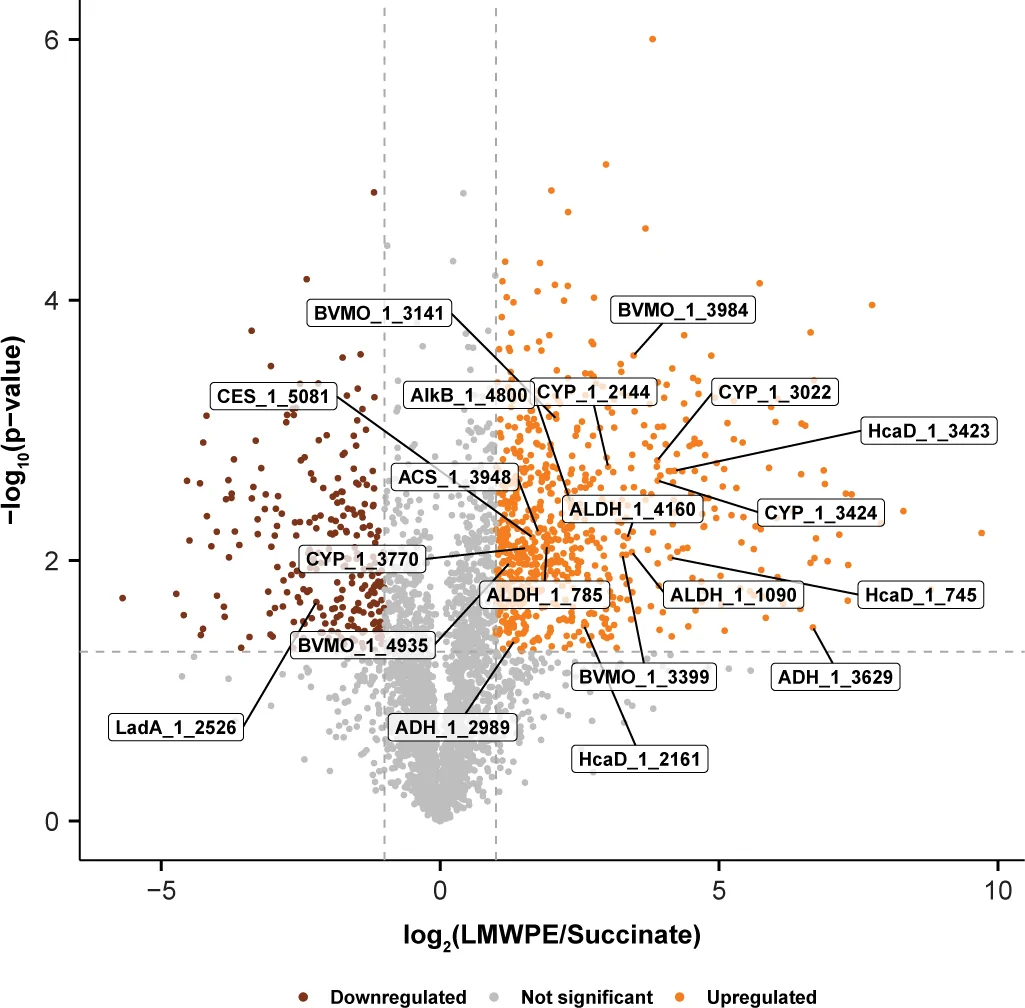
Differentially abundant proteins associated with alkane and 2-ketone degradation detected in the LMWPE-derived proteome of *Rhodococcus* sp. ASF-10. KEGG Orthology IDs, as well as BLAST hits, were used to identify and highlight proteins known or hypothesized to be involved in alkane and 2-ketone utilization. The dashed horizontal line denotes the *P* value cut-off (*P* < 0.05), and the dashed vertical lines denote the log_2_ fold-change cut-off (<−1 and >1). Source data is provided in Table S3A.

Although *Rhodococcus* sp. ASF-10 was unable to utilize the higher molecular weight fraction of LMWPE, it could nonetheless interact with PE-derived compounds. To investigate the cellular response under conditions resembling oxidized PE, we carried out a proteomic study using oxidized LMWPE as a model substrate. To identify candidate enzymes involved in the degradation of the LMWPE components, we compared the proteome of *Rhodococcus* sp. ASF-10 grown on this substrate and a succinate reference. We focused on enzymes which have been proposed in previous studies with *Rhodococcus* isolates as being able to depolymerize PE, which belong to different oxidoreductase families, such as alkane hydroxylases and laccases (17, 20, 73). Proteins homologous to those previously shown to be involved in both terminal and subterminal alkane oxidation pathways (74) were markedly more abundant in the LMWPE-derived proteome than in the succinate-derived proteome (Fig. 6). These proteins include the alkane 1-monooxygenase AlkB (RhASF-10_1_4800), a cytochrome P450 alkane hydroxylase CYP153 (RhASF-10_1_3424), multiple putative cytochrome P450s (CYP: RhASF-10_1_2144, RhASF-10_1_3022, and RhASF-10_1_3770), as well as several alcohol dehydrogenases and aldehyde dehydrogenases (Fig. 6). Alkanes are transported into the cell during the first step of the alkane degradation pathway, where AlkB hydroxylates the alkane as the compound is translocated through the cell membrane (75). An additional monooxygenase, which can catalyze degradation of long-chain alkanes (AlmA), was detected in the LMWPE proteome (RhASF-10_1_3897, log_2_ fold-change = 1.29, *P* = 0.07, Table S3A), indicating its possible involvement in the processing of long-chain alkanes by *Rhodococcus* sp. ASF-10.

Four predicted flavin-binding monooxygenases (FMOs) were also identified as differentially abundant in the LMWPE-derived proteome of *Rhodococcus* sp. ASF-10. FMOs belong to a family of xenobiotic-metabolizing enzymes, which can convert a diverse range of substrates. The family includes Baeyer–Villiger monooxygenases (BVMOs), enzymes involved in the subterminal oxidation of alkanes (76), where they oxidize carbonyl groups into an ester (74). We predicted the 3D structures of the putative *Rhodococcus* sp. ASF-10’s FMOs using AlphaFold3, which matched known BVMOs in the AFDB–SwissProt database (Fig. S1 and Table S3B). Overall, the presence of multiple differentially abundant BVMOs (RhASF-10_1_3141, RhASF-10_1_3399, RhASF-10_1_3984, and RhASF-10_1_4935) in the LMWPE proteome of *Rhodococcus* sp. ASF-10 supports their potential involvement in oxidizing 2-ketones present in the substrate into esters. Among the differentially abundant proteins, a carboxylesterase (CES, RhASF-10_1_5081) was also detected (Fig. 6). This enzyme might be involved in the hydrolysis of esters with formation of a primary alcohol and an acetate. The primary alcohol can be further metabolized by an alcohol dehydrogenase and an aldehyde dehydrogenase into a fatty acid, while the acetate can directly enter the tricarboxylic acid cycle (64). An acetyl-CoA synthetase (ACS, RhASF-10_1_3948) was also more abundant in the LMWPE proteome, suggesting that acetate produced from ester hydrolysis could be converted to acetyl-CoA (Fig. 6). Following catabolism of the primary alcohol, intermediates can be funneled into the pathway for β-oxidation of fatty acids. Indeed, enzymes involved in the β-oxidation pathway were also differentially abundant in the LMWPE-derived proteome, namely acyl-CoA dehydrogenase (ACD, RhASF-10_1_4907), long-chain acyl-CoA dehydrogenase (ACADL, RhASF-10_1_1712), enoyl-CoA hydratase (EchA, RhASF-10_1_2812), 3-hydroxyacyl-CoA dehydrogenase (FadJ, RhASF-10_1_4040), and β-ketothiolase (FadA, RhASF-10_1_1777) (Table S3A).

The levels of the enzymes involved in the pathway for 2-ketone metabolism were higher in the LMWPE proteome, with uptake of these compounds likely occurring via passive diffusion through the cell envelope (77). Fatty acid transport systems, such as lipid-transfer protein, or more unspecific hydrophobic transport complexes (78) or ABC transporters (79) may facilitate uptake. While specific transporters of methyl ketones have not been identified or characterized in literature, multiple ABC transporters were significantly differentially abundant in the LMWPE proteome (Table S3A).

While a protein (RhASF-10_2_184) exhibiting high similarity to a laccase previously reported to be PE-oxidizing (LMCO2, accession number: AII11185.1) was found in the BLAST analysis (Fig. 4) (20), it was not differentially abundant in the LMWPE-derived proteome of *Rhodococcus* sp. ASF-10 (Table S3A). If this enzyme was truly active on PE polymers, a shift in molar mass distribution of the higher M_w_ region of the LMWPE would be observed. Such a change, however, was not detected in our analysis (Fig. 5).

Overall, the proteomics results align with the analytical data of the spent LMWPE particles, demonstrating that *Rhodococcus* sp. ASF-10 can metabolize alkanes and their oxidized derivatives originating from PE. Consistently, we confirmed that *Rhodococcus* sp. ASF-10 grows on a representative alkane and 2-ketone, namely tricosane (C23 alkane) and 2-hexadecanone (C16 2-ketone) (Fig. S2).

### Analysis of co-expressed proteins reveals the involvement of biofilm and biosurfactants in LMWPE degradation

**Fig 7:**
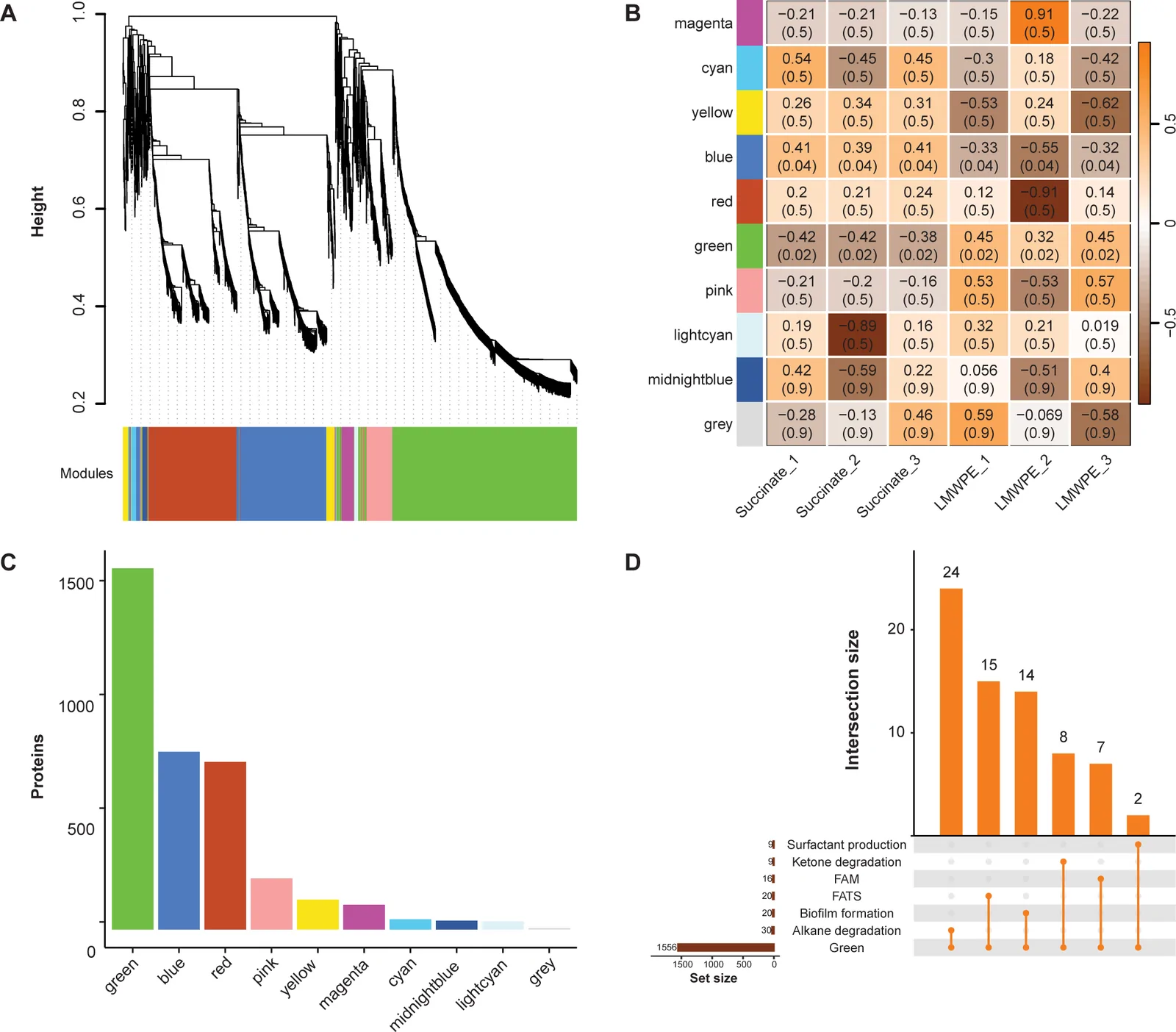
Weighted gene co-expression network analysis of *Rhodococcus* sp. ASF-10 was grown in LMWPE and succinate. (**A**) Cluster dendrogram of all proteins. (**B**) Heatmap of module eigengenes per sample. The number on top depicts the eigengene value, while the numbers between parentheses indicate the module-trait significance (*P* value). (**C**) Distribution of protein counts per module. (**D**) Upset plot with the intersections between the proteins in the green module and proteins related to biofilm formation, fatty acid transporter systems (FATS), fatty acid metabolism (FAM), surfactant production, and degradation of alkanes and 2-ketones. Source data is provided in Table S4.

To elucidate correlations between the protein abundance levels, a clustering tree was constructed using *Rhodococcus* sp. ASF-10’s LMWPE and succinate-derived proteomes. Proteins showing the same abundance patterns and high correlation were grouped into the same branch. Then, modules were defined by merging branches with similar abundance profiles (Fig. 7A). A total of 10 modules were defined. The module showing a significant positive correlation with the LMWPE proteome (“green module” in Fig. 7B, Corr = 0.99, *P* < 0.01) was also the biggest module, including a total of 1,556 proteins (Fig. 7C). The proteins in this module presented positive eigengene values for *Rhodococcus* sp. ASF-10 grown in LMWPE and negative for samples cultivated in succinate, indicating higher abundance in the LMWPE proteome (Fig. 7C).

Pathways containing proteins of interest were intersected with the proteins belonging to the green module (Fig. 7D). Specifically, this module contained proteins involved in the degradation of alkanes and 2-ketones, fatty acid transport systems, and β-oxidation of fatty acids. In addition, two enzymes associated with the production of surfactant (diacylglycerol O-acyltransferase/trehalose O-mycolyltransferase/mycolyltransferase Ag85 RhASF-10_1_270 and RhASF-10_1_4164) and 14 enzymes involved in biofilm formation (exopolysaccharide production protein ExoY RhASF-10_1_1, GDP mannose 4,6-dehydratase RhASF-10_1_2, GDP-L-fucose synthase RhASF-10_1_3, putative colanic acid biosynthesis glycosyltransferase WcaI RhASF-10_1_4, Glycosyltransferase Family 4 RhASF-10_1_5, Polysaccharide biosynthesis protein RhASF-10_1_6, adenylate cyclase RhASF-10_1_286, polysaccharide biosynthesis protein PslG RhASF-10_1_2711, RhASF-10_1_2768, adenylate cyclase RhASF-10_1_2778, 3′,5′-cyclic-AMP phosphodiesterase RhASF-10_1_3109, glucose-1-phosphate adenylyltransferase RhASF-10_1_3175, phenylacetate-CoA ligase RhASF-10_1_4153, and polysaccharide biosynthesis protein RhASF-10_1_5004) were detected, suggesting their possible contribution in facilitating surface attachment, emulsification, and stable colonization of LMWPE particles by *Rhodococcus* sp. ASF-10 (Table S4). Trehalose-containing glycolipids are biosurfactants produced by *Rhodococcus* spp. and other members of the Mycobacteriales order. The production of biofilm and surfactant has been previously reported during the growth of *Rhodococcus* spp. on *n-*alkanes (80, 81). Biosurfactants can aid in substrate accessibility, particularly during the degradation of hydrophobic water-insoluble substrates (82), such as in the case of hydrophobic alkanes and 2-ketones. Attachment of bacterial cells onto the LMWPE surface through biofilm formation could also aid in substrate accessibility through closer proximity to the hydrophobic PE derivatives.

Of note, the *Rhodococcus* sp. ASF-10’s LMCO (RhASF-10_2_184), which is highly similar to an enzyme previously reported to mediate PE degradation (20), was not detected in the LMWPE-associated module. These findings further suggest that this enzyme is unlikely to be involved in the degradation of polymeric PE, nor the PE derivatives present in the oxidized LMWPE substrate.

### Activity of *Rhodococcus* sp. ASF-10 in the gut of freshwater-raised Atlantic salmon

We investigated the activity of *Rhodococcus* sp. ASF-10 in the salmon gut using a metatranscriptomic data set generated in a previous study (13). These samples were obtained from the guts of parr, pre-smolts, and smolts, all of which are developmental stages of Atlantic salmon in freshwater. Reads were pseudo-aligned against the SMGA (11), supplemented with gene annotations for *Rhodococcus* sp. ASF-10. ASF-10 was metabolically active in the gut of fish at all three developmental stages (Fig. S3). Remarkably, some genes for alkane, 2-ketone, and fatty acid metabolism were found to be expressed under *in vivo* conditions (Fig. S4), in line with the proteomics results (Fig. 6). The genes for alkane and ketone utilization include *cyp* (RhASF-10_1_3022; Cytochrome P450), *cyp153s* (RhASF-10_1_4337 and RhASF-10_1_3424; Cytochrome P450), *almA* (RhASF-10_1_3897; AlmA monooxygenase), *adh* (RhASF-10_1_3629; alcohol dehydrogenase), *acsl* (RhASF-10_1_3121; long-chain acyl-CoA synthetase), *aldh* (RhASF-10_1_1090; aldehyde dehydrogenase), and *acs* (RhASF-10_1_3948; acetyl-CoA synthetase) (Fig. S4). The metatranscriptomic data originate from freshwater salmon fed commercial diets, and PE and/or PE derivatives may be present in the tanks or in MPs in the feed. To be available for utilization by salmon gut bacteria, PE and/or its derivatives must pass through the digestive system of the Atlantic salmon. The stomach environment of freshwater juveniles is mildly acidic (pH 5.1), while the intestine is mildly alkaline (pH 8.3–8.6) (83). At physiological pH, the chemically inert alkanes and stable 2-ketones (84) are expected to pass through the salmon gut largely unaltered, where bacteria, such as *Rhodococcus*, commonly found in the Atlantic salmon gut (58), can utilize these hydrocarbons. While the occurrence of *Rhodococcus* sp. ASF-10 in the salmon gut is not dependent on the availability of hydrocarbons, these compounds may serve as a carbon source under conditions where oxidized PE pollution is present in feed or the surrounding environment.

### Concluding remarks

In this study, we systematically investigated the genomic basis for hydrocarbon decomposition by *Rhodococcus* sp. ASF-10 within the context of current knowledge on *Rhodococcus* spp., followed by proteomics-based identification of candidate enzymes employed by ASF-10 to mineralize molecules derived from oxidized LMWPE. The distillation of functional annotations into metabolic traits revealed a gradient of metabolic capacities among *Rhodococcus* spp., which correlated with their genome sizes up to 6 Mb, and were independent of the isolation source (Fig. 3). Alkane utilization emerged as a core function among all the *Rhodococcus* genomes analyzed (Fig. 4). In-depth analytical and proteomic insight demonstrated that *Rhodococcus* sp. ASF-10 can selectively metabolize alkanes with carbon length up to C29 and 2-ketones up to C28 when growing in LMWPE, while the polymeric fraction of this substrate remained undegraded (Fig. 5). Proteomic analysis and WGCNA showed that this bacterium utilizes a repertoire of alkane and 2-ketone degrading enzymes, such as AlkB and BVMOs, and produces biosurfactants and biofilms in its metabolism of LMWPE (Fig. 6 and 7). Remarkably, proteins previously indicated to be involved in PE utilization, including laccases and LMCOs, by other *Rhodococcus* isolates were not differentially abundant in our proteomic analysis. This suggests that the actual breakdown of the polymeric component by these enzymes may need to be reassessed. The importance of combining advanced substrate characterization (for instance using analytical techniques, such as SEC and GC-MS) before and after bacterial growth to accurately determine whether microbes can cleave the PE backbone and subsequently mineralize it cannot be exaggerated, especially as the field of plastic degradation is increasingly searching for microbes and their enzymes to tackle this global pollution problem.

Finally, our study highlights the metabolic activity of *Rhodococcus* sp. ASF-10 in the gut of freshwater-reared salmon across different life stages, with enzymatic features linked to alkane, ketone, and fatty acid utilization, suggesting a biologically relevant role in hydrocarbon turnover *in vivo* (Fig. S3 and S4).

Overall, our findings provide significant insights into the capability of the salmon gut bacterium *Rhodococcus* sp. ASF-10 to metabolize alkanes and 2-ketones from abiotically oxidized LMWPE. Although these results are based on controlled *in vitro* experiments, metatranscriptomic analyses provide supporting evidence for the biological relevance of *Rhodococcus* sp. ASF-10 in these metabolic processes in the salmon gut. While further *in vivo* validation is required, our findings open venues to potentially exploit this microbe and its enzymes as a bioremediation tool for hydrocarbons and microplastic derivatives in aquaculture settings.

## Data Availability

The genome sequence, gene annotations, and predicted proteins have been made publicly available via FigShare (https://doi.org/10.6084/m9.figshare.30731261). The mass spectrometry proteomics data have been deposited in the ProteomeXchange Consortium via the PRIDE (Proteomics Identification Database) partner repository with the data set identifier PXD069510. Scripts used to generate the phylogenetic tree, heatmap, volcano plot, and WGCNA, can be found at https://github.com/ronjasan/RhASF-10. All data are publicly available as of the date of publication and comply with the data reuse guidelines presented in Hug et al. (85).

## SUPPLEMENTAL MATERIAL

Supplemental figures aem.00174-26-s0001.pdf Fig. S1 to S4.

Table S1 aem.00174-26-s0002.xlsx BLASTp hits in the predicted proteomes of *Rhodococcus* spp. against protein sequences obtained from PlasticDB, PAZy, and HADEG databases.

Table S2 aem.00174-26-s0003.xlsx Source data for FT-IR, SEC, and GC-MS.

Table S3 aem.00174-26-s0004.xlsx Source data for proteomics, as well as AlphaFold3 structure predictions for the BVMOs in *Rhodococcus* sp. ASF-10 that were detected as differentially abundant in the LMWPE proteome.

Table S4 aem.00174-26-s0005.xlsx Source data for the WGCNA.

Table S5 aem.00174-26-s0006.xlsx Expression of *Rhodococcus* sp. ASF-10 genes encoding proteins putatively involved in alkane, 2-ketone, and degradation of various plastics.

ASM does not own the copyrights to Supplemental Material that may be linked to, or accessed through, an article. The authors have granted ASM a non-exclusive, world-wide license to publish the Supplemental Material files. Please contact the corresponding author directly for reuse.
