# Supplementary material for: Biodegradation of components from an oxidized polyethylene by a Rhodococcus strain isolated from the gut of Atlantic salmon
Source: Appl Environ Microbiol. 2026 Jul 10;92(8):e00174-26. doi: 10.1128/aem.00174-26 (PMC13488340; doi:10.1128/aem.00174-26)
Supplement: Supplementary file 1 [file aem.00174-26-s0001.pdf]

Supplemental Materials for

Biodegradation of components from an oxidized polyethylene by a *Rhodococcus* strain isolated from the gut of Atlantic Salmon

Ronja Marlonsdotter Sandholm[a], Dave Edison Rojas Calderon[a], Marcus Torres Hansen[a], Ravindra R. Chowreddy[b], Gustav Vaaje-Kolstad[a], Sabina Leanti La Rosa[a#]

[a] Faculty of Chemistry, Biotechnology and Food Science, Norwegian University of Life Sciences, Ås, Norway.

[b] Norner Research AS, Porsgrunn, Norway

[#]Corresponding author: Sabina Leanti La Rosa, sabina.leantilarosa@nmbu.no

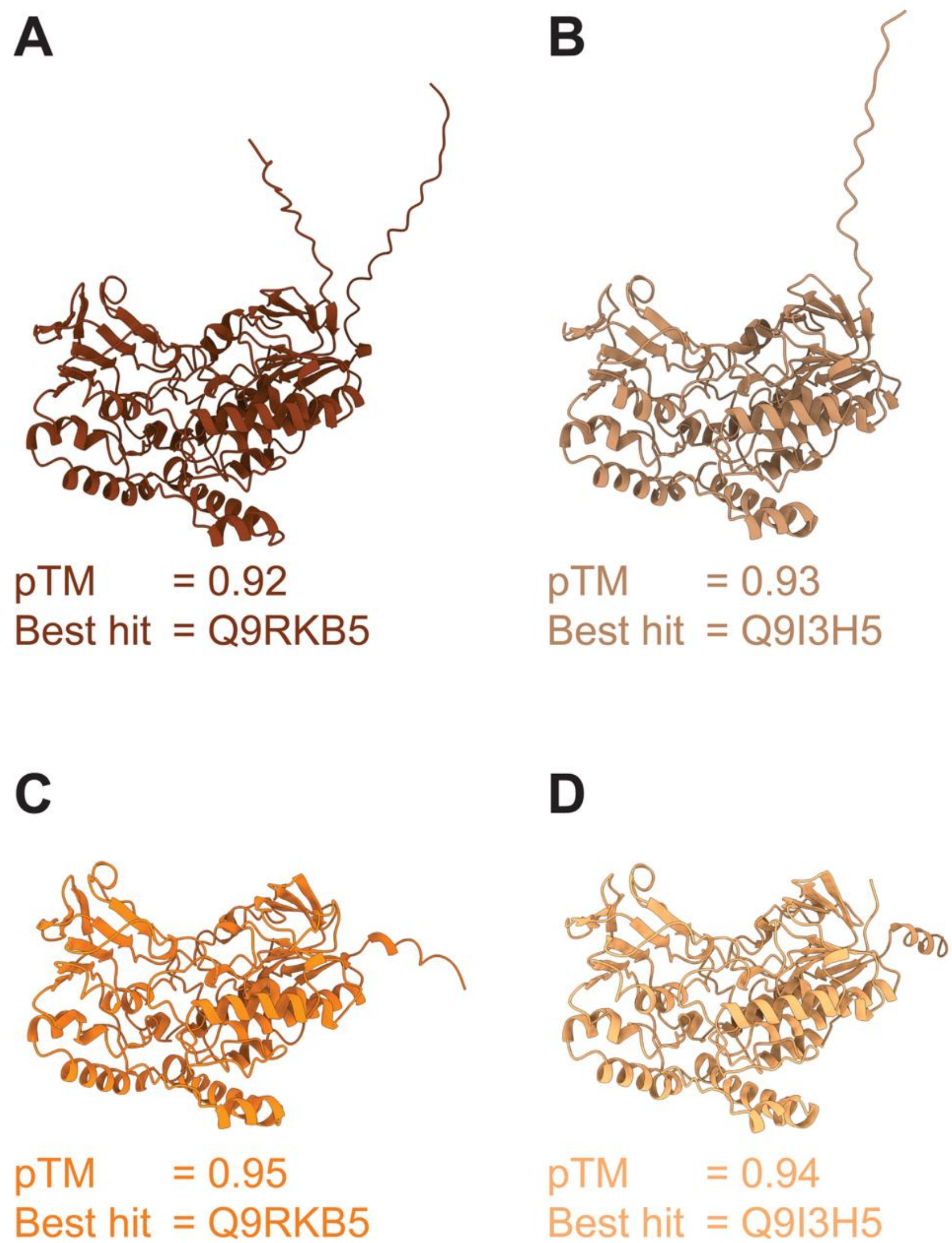

Figure S1. Predicted AlphaFold3 structures for the differentially abundant BVMOs detected in the *Rhodococcus* sp. ASF-10 proteomics data. **(A)** BVMO_1_3141, **(B)** BMVO_1_3399, **(C)** BVMO_1_3984, and **(D)** BVMO_1_4935. AlphaFold3 structure prediction scores (pTM) and UniProt accession number for the Foldseek best hit in the AFDB-SWISSPROT database are displayed in the figure, while e-value and amino acid sequence identity are provided in **Table S3B**.

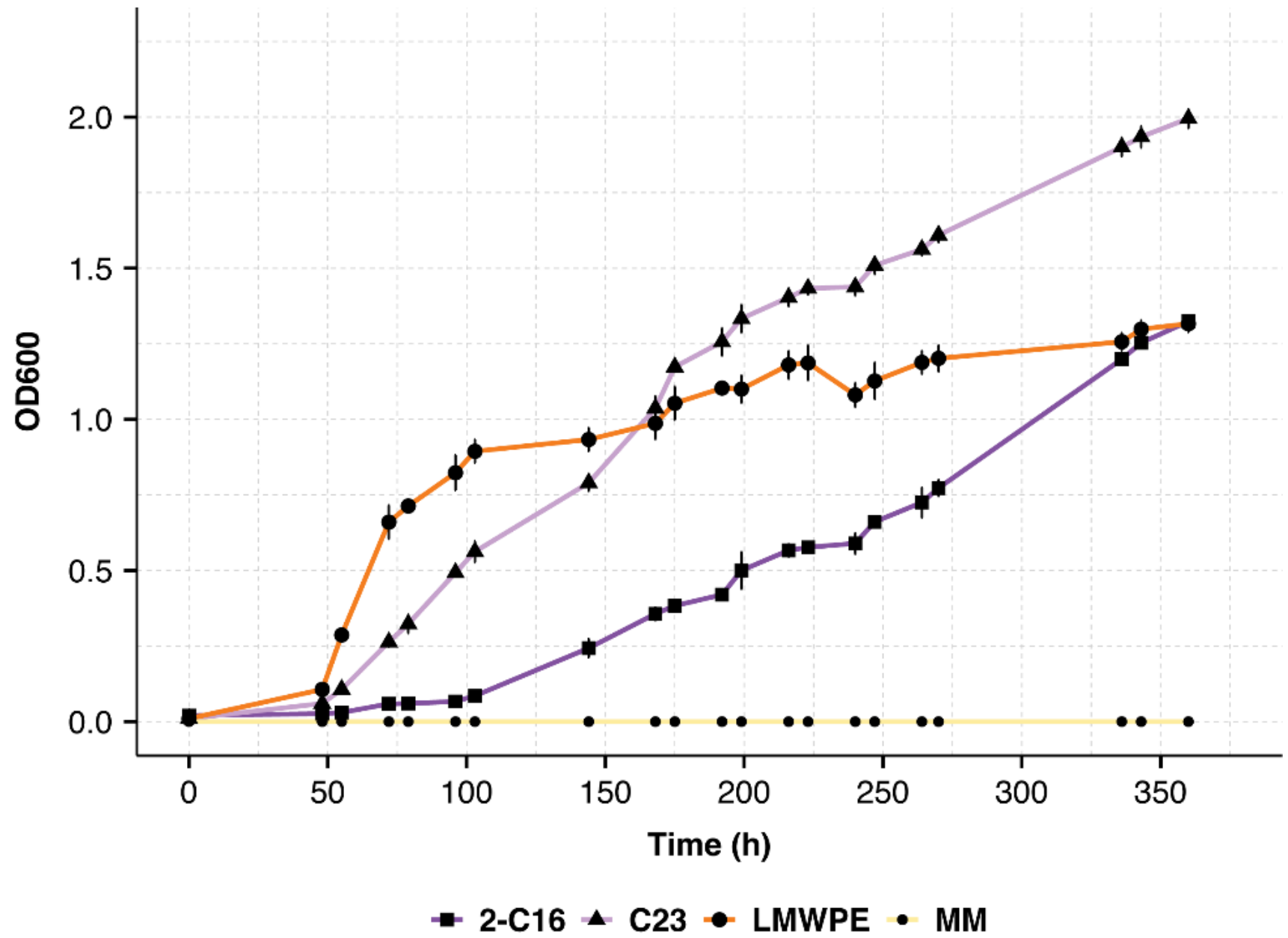

Figure S2. Growth curves of *Rhodococcus* sp. ASF-10 at 22 °C in minimal medium supplemented with 30 mg/mL tricosane, 2-hexadecanone, or LMWPE. Growth was measured as the optical density at 600 nm (OD600). The values represent the averages of two biological replicates with technical triplicates. Abbreviations: C23, tricosane (alkane); 2-C16, 2-hexadecanone (2-ketone); LMWPE, low-molecular-weight PE; MM, minimal medium no carbon source.

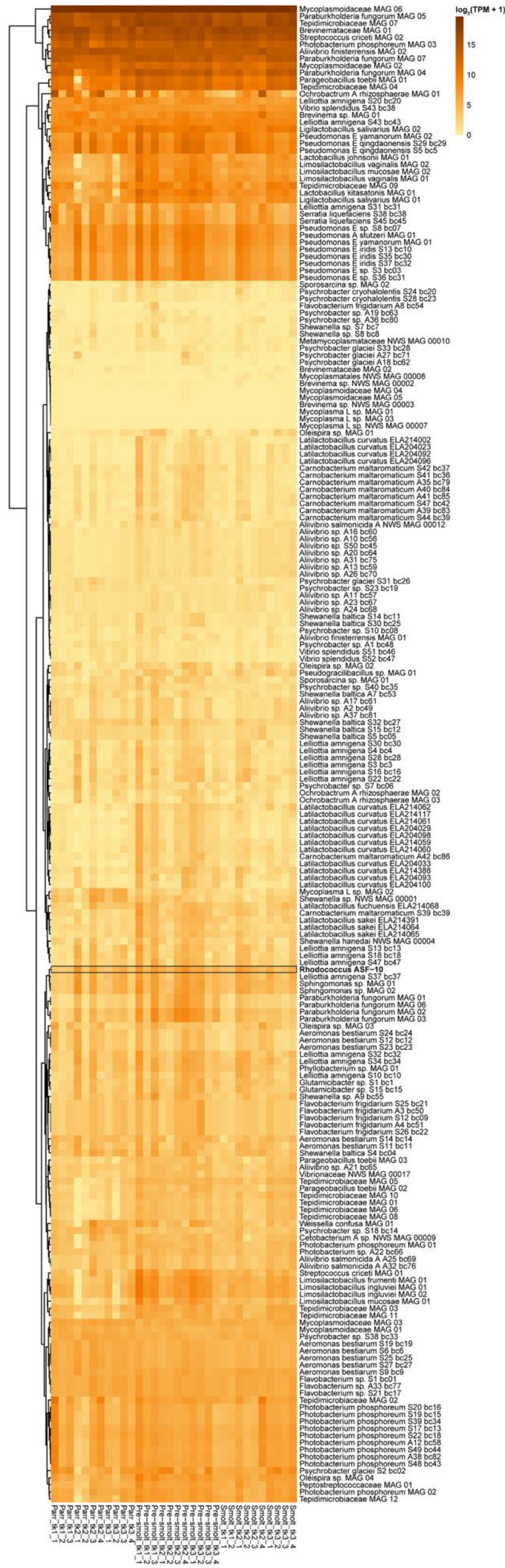

log2(TPM + 1)
15
10
5
0
Mycoplasmoidaceae MAG 06
Paraburkholderia fungorum MAG 05
Tepidimicrobiaceae MAG 07
Brevinemataceae MAG 01
Streptococcus criceti MAG 02
Photobacterium phosphoreum MAG 03
Aliivibrio finisterrensis MAG 02
Paraburkholderia fungorum MAG 07
Mycoplasmoidaceae MAG 02
Paraburkholderia fungorum MAG 04
Parageobacillus toebii MAG 01
Tepidimicrobiaceae MAG 04
Ochrobactrum A rhizosphaerae MAG 01
Lelliottia amnigena S20 bc20
Vibrio splendidus S43 bc38
Brevinema sp. MAG 01
Lelliottia amnigena S43 bc43
Ligilactobacillus salivarius MAG 02
Pseudomonas E yamanorum MAG 02
Pseudomonas E qingdaonensis S29 bc29
Pseudomonas E qingdaonensis S5 bc5
Lactobacillus johnsonii MAG 01
Limosilactobacillus vaginalis MAG 02
Limosilactobacillus mucosae MAG 02
Limosilactobacillus vaginalis MAG 01
Tepidimicrobiaceae MAG 09
Lactobacillus kitasatonis MAG 01
Ligilactobacillus salivarius MAG 01
Lelliottia amnigena S31 bc31
Serratia liquefaciens S38 bc38
Serratia liquefaciens S45 bc45
Pseudomonas E sp. S8 bc07
Pseudomonas A stutzeri MAG 01
Pseudomonas E yamanorum MAG 01
Pseudomonas E iridis S13 bc10
Pseudomonas E iridis S35 bc30
Pseudomonas E iridis S37 bc32
Pseudomonas E sp. S3 bc03
Pseudomonas E sp. S36 bc31
Sporosarcina sp. MAG 02
Psychrobacter cryohalolentis S24 bc20
Psychrobacter cryohalolentis S28 bc23
Flavobacterium frigidarium A8 bc54
Psychrobacter sp. A19 bc63
Psychrobacter sp. A36 bc80
Shewanella sp. S7 bc7
Shewanella sp. S8 bc8
Metamycoplasmataceae NWS MAG 00010
Psychrobacter glaciei S33 bc28
Psychrobacter glaciei A27 bc71
Psychrobacter glaciei A18 bc62
Brevinemataceae MAG 02
Mycoplasmatales NWS MAG 00008
Brevinema sp. NWS MAG 00002
Mycoplasmoidaceae MAG 04
Mycoplasmoidaceae MAG 05
Brevinema sp. NWS MAG 00003
Mycoplasma L sp. MAG 01
Mycoplasma L sp. MAG 03
Mycoplasma L sp. NWS MAG 00007
Oleispira sp. MAG 01
Latilactobacillus curvatus ELA214002
Latilactobacillus curvatus ELA204023
Latilactobacillus curvatus ELA204092
Latilactobacillus curvatus ELA204096
Carnobacterium maltaromaticum S42 bc37
Carnobacterium maltaromaticum S41 bc36
Carnobacterium maltaromaticum A35 bc79
Carnobacterium maltaromaticum A40 bc84
Carnobacterium maltaromaticum A41 bc85
Carnobacterium maltaromaticum S47 bc42
Carnobacterium maltaromaticum A39 bc83
Carnobacterium maltaromaticum S44 bc39
Aliivibrio salmonicida A NWS MAG 00012
Aliivibrio sp. A16 bc60
Aliivibrio sp. A10 bc56
Aliivibrio sp. S50 bc45
Aliivibrio sp. A20 bc64
Aliivibrio sp. A31 bc75
Aliivibrio sp. A13 bc59
Aliivibrio sp. A26 bc70
Psychrobacter glaciei S31 bc26
Psychrobacter sp. S23 bc19
Aliivibrio sp. A11 bc57
Aliivibrio sp. A23 bc67
Aliivibrio sp. A24 bc68
Shewanella baltica S14 bc11
Shewanella baltica S30 bc25
Psychrobacter sp. S10 bc08
Aliivibrio finisterrensis MAG 01
Psychrobacter sp. A1 bc48
Vibrio splendidus S51 bc46
Vibrio splendidus S52 bc47
Oleispira sp. MAG 02
Pseudogracilibacillus sp. MAG 01
Sporosarcina sp. MAG 01
Psychrobacter sp. S40 bc35
Shewanella baltica A7 bc53
Aliivibrio sp. A17 bc61
Aliivibrio sp. A2 bc49
Aliivibrio sp. A37 bc81
Shewanella baltica S32 bc27
Shewanella baltica S15 bc12
Shewanella baltica S5 bc05
Lelliottia amnigena S30 bc30
Lelliottia amnigena S4 bc4
Lelliottia amnigena S28 bc28
Lelliottia amnigena S3 bc3
Lelliottia amnigena S16 bc16
Lelliottia amnigena S22 bc22
Psychrobacter sp. S7 bc06
Ochrobactrum A rhizosphaerae MAG 02
Ochrobactrum A rhizosphaerae MAG 03
Latilactobacillus curvatus ELA214062
Latilactobacillus curvatus ELA214117
Latilactobacillus curvatus ELA214061
Latilactobacillus curvatus ELA204029
Latilactobacillus curvatus ELA204098
Latilactobacillus curvatus ELA214059
Latilactobacillus curvatus ELA214060
Carnobacterium maltaromaticum A42 bc86
Latilactobacillus curvatus ELA204033
Latilactobacillus curvatus ELA214388
Latilactobacillus curvatus ELA204093
Latilactobacillus curvatus ELA204100
Mycoplasma L sp. MAG 02
Shewanella sp. NWS MAG 00001
Latilactobacillus fuchuensis ELA214068
Carnobacterium maltaromaticum S39 bc39
Latilactobacillus sakei ELA214391
Latilactobacillus sakei ELA214064
Latilactobacillus sakei ELA214065
Shewanella hanedai NWS MAG 00004
Lelliottia amnigena S13 bc13
Lelliottia amnigena S18 bc18
Lelliottia amnigena S47 bc47
Rhodococcus ASF−10
Lelliottia amnigena S37 bc37
Sphingomonas sp. MAG 01
Sphingomonas sp. MAG 02
Paraburkholderia fungorum MAG 01
Paraburkholderia fungorum MAG 06
Paraburkholderia fungorum MAG 02
Paraburkholderia fungorum MAG 03
Oleispira sp. MAG 03
Aeromonas bestiarum S24 bc24
Aeromonas bestiarum S12 bc12
Aeromonas bestiarum S23 bc23
Lelliottia amnigena S32 bc32
Lelliottia amnigena S34 bc34
Phyllobacterium sp. MAG 01
Lelliottia amnigena S10 bc10
Glutamicibacter sp. S1 bc1
Glutamicibacter sp. S15 bc15
Shewanella sp. A9 bc55
Flavobacterium frigidarium S25 bc21
Flavobacterium frigidarium A3 bc50
Flavobacterium frigidarium S12 bc09
Flavobacterium frigidarium A4 bc51
Flavobacterium frigidarium S26 bc22
Aeromonas bestiarum S14 bc14
Aeromonas bestiarum S11 bc11
Shewanella baltica S4 bc04
Parageobacillus toebii MAG 03
Aliivibrio sp. A21 bc65
Vibrionaceae NWS MAG 00017
Tepidimicrobiaceae MAG 05
Parageobacillus toebii MAG 02
Tepidimicrobiaceae MAG 10
Tepidimicrobiaceae MAG 01
Tepidimicrobiaceae MAG 06
Tepidimicrobiaceae MAG 08
Weissella confusa MAG 01
Psychrobacter sp. S18 bc14
Cetobacterium A sp. NWS MAG 00009
Photobacterium phosphoreum MAG 01
Photobacterium sp. A22 bc66
Aliivibrio salmonicida A A25 bc69
Aliivibrio salmonicida A A32 bc76
Streptococcus criceti MAG 01
Limosilactobacillus frumenti MAG 01
Limosilactobacillus ingluviei MAG 01
Limosilactobacillus ingluviei MAG 02
Limosilactobacillus mucosae MAG 01
Tepidimicrobiaceae MAG 03
Tepidimicrobiaceae MAG 11
Mycoplasmoidaceae MAG 03
Mycoplasmoidaceae MAG 01
Psychrobacter sp. S38 bc33
Aeromonas bestiarum S19 bc19
Aeromonas bestiarum S6 bc6
Aeromonas bestiarum S25 bc25
Aeromonas bestiarum S27 bc27
Aeromonas bestiarum S9 bc9
Flavobacterium sp. S1 bc01
Flavobacterium sp. A33 bc77
Flavobacterium sp. S21 bc17
Tepidimicrobiaceae MAG 02
Photobacterium phosphoreum S20 bc16
Photobacterium phosphoreum S19 bc15
Photobacterium phosphoreum S39 bc34
Photobacterium phosphoreum S17 bc13
Photobacterium phosphoreum S22 bc18
Photobacterium phosphoreum A12 bc58
Photobacterium phosphoreum S49 bc44
Photobacterium phosphoreum A38 bc82
Photobacterium phosphoreum S48 bc43
Psychrobacter glaciei S2 bc02
Oleispira sp. MAG 04
Peptostreptococcaceae MAG 01
Photobacterium phosphoreum MAG 02
Tepidimicrobiaceae MAG 12
Parr_tk1_1
Parr_tk1_2
Parr_tk1_3
Parr_tk2_1
Parr_tk2_2
Parr_tk2_3
Parr_tk2_4
Parr_tk3_1
Parr_tk3_2
Parr_tk3_3
Parr_tk3_4
Pre−smolt_tk1_1
Pre−smolt_tk1_2
Pre−smolt_tk1_3
Pre−smolt_tk2_1
Pre−smolt_tk2_2
Pre−smolt_tk2_3
Pre−smolt_tk2_4
Pre−smolt_tk3_1
Pre−smolt_tk3_2
Pre−smolt_tk3_3
Pre−smolt_tk3_4
Smolt_tk1_1
Smolt_tk1_2
Smolt_tk2_1
Smolt_tk2_2
Smolt_tk2_3
Smolt_tk2_4
Smolt_tk3_1
Smolt_tk3_2
Smolt_tk3_3
Smolt_tk3_4

Figure S3. Heatmap showing gene expression patterns in salmon gut metatranscriptomes. Metatranscriptomic samples from freshwater Atlantic salmon (parr, pre-smolt, and smolt stages) were obtained from Gupta *et al.* 2024 (PMID: 39455736). Raw metatranscriptomic reads were mapped against all genomes and MAGs in the SMGA (PMID: 39402236), supplemented with *Rhodococcus* sp. ASF-10 (highlighted in bold). Expression values are shown as $\log_2$-transformed Transcripts Per Million (TPM+1). Source data are provided via FigShare (https://doi.org/10.6084/m9.figshare.32038314). Abbreviation: Tk = tank.

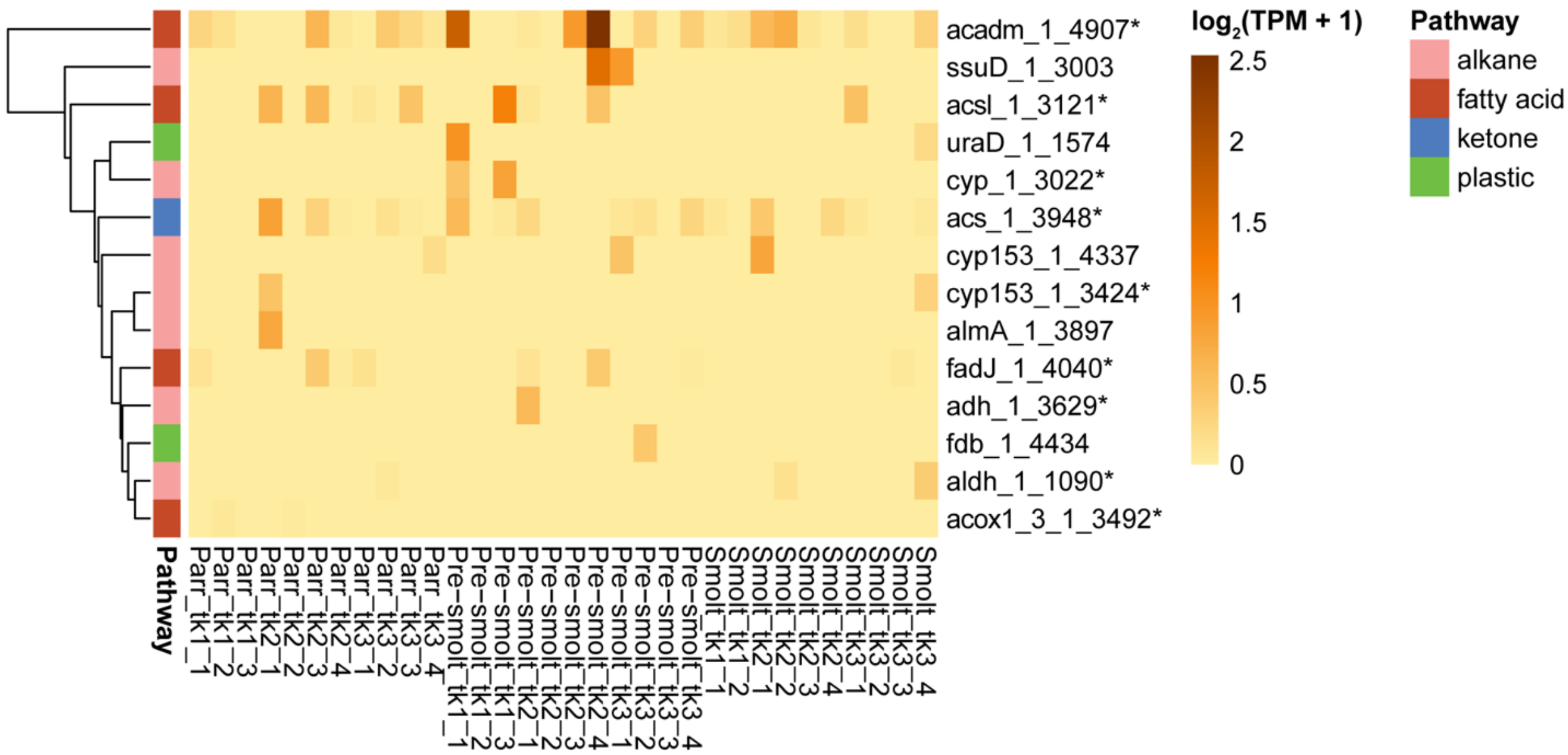

Figure S4. Transcriptional status of *Rhodococcus* sp. ASF-10's genes encoding proteins involved in alkane, 2-ketone and fatty acid utilization as well as genes coding for proteins possibly involved in plastic degradation. Genes marked with an asterisk correspond to differentially abundant proteins detected in the LMWPE proteome (Figure 6). Expression values are shown as log_2-transformed Transcripts Per Million (TPM + 1). Abbreviations: Tk = tank; acadm, acyl-CoA dehydrogenase; ssuD, alkanesulfonate monooxygenase; acsl, long-chain acyl-CoA synthetase; uraD, allantoinase; cyp, Cytochrome P450; acs, long-chain acyl-CoA synthetase; cyp153, Cytochrome P450; almA, monooxygenase; fadJ, 3-hydroxyacyl-CoA dehydrogenase; adh, alcohol dehydrogenase; fdb, non-heme chloroperoxidase; aldh, aldehyde dehydrogenase; acox, acyl-CoA oxidase. Source data for this figure is provided in **Table S5**.
